# Can qualia be geometric? A geometro-diffusion framework for the relational structure of color experience

**DOI:** 10.1007/s11571-026-10577-2

**Published:** 2026-11-07

**Authors:** Ernesto Estrada

**Affiliations:** Institute for Cross-Disciplinary Physics and Complex Systems (IFISC), CSIC-UIB, Palma de Mallorca, Spain

**Keywords:** Geometro-Diffusion Theory, Consciousness, Qualia, Diffusion-induced geometry, Color perception, Human connectome

## Abstract

Within the Geometro-Diffusion Theory (GDT) of consciousness, we formulate a candidate geometry for the relational organization of color experience. We distinguish a diffusion-induced scaffold, the color content encoded on it, and its neuronal realization. Elementary network examples establish these distinctions and show how consensus-directed nonlinear activity preserves the diffusion scaffold. On a 76-region human structural connectome, a distributed encoding is learned from 16 controlled colors and then frozen. Evaluation on 256 new colors gives a Spearman correspondence of 0.919 with the CIEDE2000 perceptual reference, compared with 0.923 for direct CIELAB distances. Nonlinear Izhikevich encoding followed by the same anatomical propagation gives analytic–neuronal correspondence of 0.924. The learned construction therefore transfers color relations to new stimuli and expresses much of their geometric organization through nonlinear neuronal activity. Connections with predictive coding and cortical gradients identify possible roles for the geometry of anticipated and stimulus-evoked responses. Within GDT, intrinsic access and recursive use link this relational organization to qualitative experience. The results provide a mathematical and computational candidate for that organization, together with explicit empirical comparisons for evaluating its experiential interpretation.

**Supplementary Information:**

The online version contains supplementary material available at https://doi.org/10.1007/s11571-026-10577-2.

## Introduction

Consciousness is commonly characterized by subjective experience: the felt colorness of a color, painfulness of pain, or auditory quality of a sound. In the distinction developed here, observation concerns detecting and discriminating signals, whereas experience concerns their felt qualitative character. Such phenomenal qualities, often termed qualia, motivate the question of how physical activity relates to experience (Koch 2018; Forti 2025; Kauffman and Roli 2023). The hard problem concerns why physical processes should be accompanied by experience at all (David and Chalmers 1997). A related but distinct scientific question concerns the relational organization of color representations: how similarities and differences among colors are structured at the observational level. The present paper develops a geometric account of this organization within the Geometro-Diffusion Theory (GDT).

Contemporary theories of consciousness emphasize broadcasting, causal integration, recurrence, inference, and higher-order representation in different ways (Dehaene et al. 2017; Seth and Bayne 2022). The search for neural correlates also distinguishes experience from report, attention, and behavioural response (Koch et al. 2016). Nonlinear and complex-systems approaches relate consciousness to collective dynamics, criticality, and phase-space organization (Werner 2013, 2009; Yurchenko 2023; Allegrini et al. 2013; Tegmark 2015; Steven Ravett Brown 2013). These approaches motivate studying distributed physical organization and formulating explicit hypotheses connecting that organization to phenomenality.

Vision provides a tractable domain because stimuli, neuronal responses, and perceptual judgments can be compared. Visual awareness involves distributed and temporally organized interactions across cortical stages (Crick and Koch 1995; Rees et al. 2002; Tong 2003; Victor and Lamme 2006). color is especially useful: lightness, chroma, and hue vary continuously, while similarity and category relations structure the perceptual domain. Chromatic processing extends across early visual, extrastriate, and ventral occipitotemporal regions (Gegenfurtner 2003; Conway et al. 2007; Lafer-Sousa and Conway 2013), and color information can be decoded from distributed human cortical activity (Brouwer and Heeger 2009).

GDT begins with a physical relational substrate supporting propagation (Estrada 2026c). A particular input generates one response, whereas the propagation operator determines a repertoire of responses whose pairwise relations define a geometry. The geometry is jointly specified by the substrate and the propagation law. In the content construction studied here, it additionally depends on the encoding map. GDT hypothesizes that qualitative organization is associated with relations within this geometry when they are intrinsically accessible and causally used (Estrada 2026a). Recurrent interactions, convergent pathways, population decoding, and temporal integration provide possible mechanisms of access (Victor and Lamme 2006; Mashour et al. 2020; Panzeri et al. 2015; Camilo Miguel Signorelli and Daniel Meling 2021; Taylor 2007). The Diffusion-Based Machine (Estrada 2026c) denotes this distributed functional capacity.

This hypothesis has substantive connections with predictive accounts. Predictive coding distinguishes internally generated expectations from the sensory discrepancies that update them Rao and Ballard (1999); Friston and Kiebel (2009). The distinction between a realized state and a dynamical repertoire suggests a corresponding interpretation of the observational and geometric levels of GDT. Connectivity-gradient studies describe continuous cortical organization (Margulies et al. 2016), and explicitly relate that organization to predictive representations and their modulation (Zhang et al. 2019; Katsumi et al. 2023). Structural connectome harmonics and predictive maps supply complementary mathematical links between network modes, possible responses, and anticipated future states (Atasoy et al. 2016; Stachenfeld et al. 2017). These connections are developed in Section 6.

GDT proposes intrinsic access as a condition connecting physical relational organization to experience. This proposal addresses the functional availability of geometric relations; the explanatory question of why such organization has subjective character remains a theoretical question. Our aim is to construct and assess a candidate relational organization of qualitative content, specify its physical realization, and formulate empirical comparisons through which the GDT hypothesis can be evaluated.

We use CIELAB coordinates and CIEDE2000 color differences as controlled psychophysical reference constructions (International Organization for Standardization and International Commission on Illumination 2019; Sharma et al. 2005). They provide standardized benchmarks for relating the constructed geometry to perceptual organization. The elementary examples distinguish a diffusion scaffold, particular color content, and dynamical neuronal decoration. We then implement distributed color encoding on a region-level structural connectome derived from the Hungarian/HCP resources (Szalkai et al. 2017; Kerepesi et al. 2017). The support comprises bilateral pericalcarine, lateral occipital, lingual, and fusiform regions.

The calculations determine how a learned encoding and anatomical propagation induce a content metric, how its ordering generalizes to new colors, and whether that organization remains expressed after nonlinear Izhikevich encoding followed by the same structural propagation (Izhikevich 2003, 2007). The resulting contribution is an anatomically grounded computational construction and a programme for testing its relation to color experience. The empirical programme evaluates how the resulting deformation relates to subjective color judgments.

## Geometro-diffusion theory in a nutshell

**Fig. 1:**
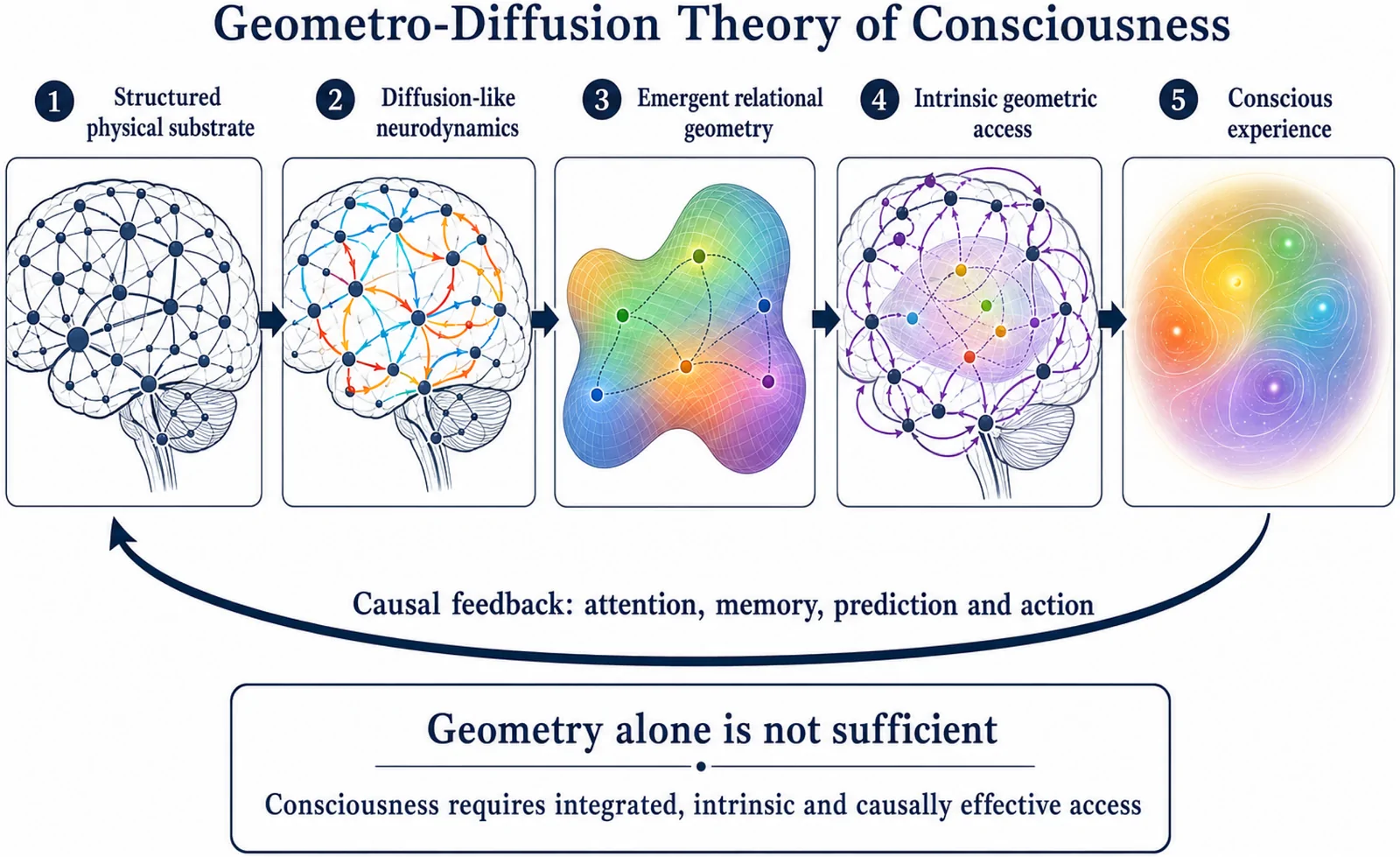
Schematic overview of the Geometro-Diffusion Theory (GDT) of consciousness. Diffusion-like neurodynamics acting on a structured physical substrate generate an emergent relational geometry in which distinct qualitative contents occupy differentiated but mutually related regions. Within GDT, conscious experience requires integrated, intrinsic, and causally effective access to the geometry generated by the system’s own dynamics. This access enables the resulting relational organization to influence attention, memory, prediction, action, and subsequent network activity through recursive causal feedback

The Geometro-Diffusion Theory (GDT) of consciousness was introduced and developed formally by Estrada (2026c). The present section provides a self-contained and pedagogical account of the concepts required in this paper. The central construction may be understood as a sequence of levels. A physical relational structure constrains the propagation of signals; the complete family of possible propagation patterns determines an intrinsic geometry; and this geometry may contain relational information accessible through the integrated response family. GDT proposes that conscious experience depends on the existence of such a geometry together with its intrinsic accessibility and causal use by the system that generates it. The main conceptual flow of the GDT of consciousness is represented in Fig. 1.

### Relational substrates and propagation dynamics

Consider a neural or biological system described, at a chosen spatial and functional scale, by a relational substrate *G=(V,E)*, where *V* is a set of *n=|V|* elements and *E* specifies the relations that constrain signal propagation. Depending on the scale of description, the vertices may represent individual neurons, neuronal populations, cortical regions, molecular compartments, or other interacting units. In the simplest formulation, *G* is a graph.

Let $\textbf{c}(t)\in \mathbb {R}^{n}$ describe the state distributed over the system at time *t*. For a linear diffusion-like process (Masuda et al. 2017; Estrada 2024), its evolution is governed by

2.1 $$\begin{aligned} \frac{d\textbf{c}(t)}{dt}=-\mathscr {L}_{\textrm{gen}}\textbf{c}(t),\qquad \textbf{c}(0)=\textbf{c}_{0}, \end{aligned}$$

and therefore

2.2 $$\begin{aligned} \textbf{c}(t)=\exp \!\left( -t\mathscr {L}_{\textrm{gen}}\right) \textbf{c}_{0}. \end{aligned}$$

The operator $\mathscr {L}_{\textrm{gen}}$ represents both the relational organization of the substrate and the dynamical constraints imposed on propagation. For conservative diffusion on an undirected weighted graph, it reduces to the combinatorial Laplacian $\mathscr {L}=\mathscr {K}-\mathscr {A},$ where $\mathscr {A}$ is the weighted adjacency matrix and $\mathscr {K}$ is the diagonal matrix of vertex strengths. Intuitively, the Laplacian encodes how strongly each element is coupled to its neighbors, so that $\exp (-t\mathscr {L}_{\textrm{gen}})$ describes how a signal placed at one element spreads to the rest of the network as *t* increases. More general choices of $\mathscr {L}_{\textrm{gen}}$ may account for directionality, anomalous propagation, production or removal of the transported quantity, leakage, clearance, heterogeneous timescales, or dependence on the current state of the system. Weighted, directed, temporal, multilayer, and higher-order systems can be incorporated by replacing the graph Laplacian with a propagation operator appropriate to the structure under consideration.

The construction extends to other physical propagation processes. A formally equivalent kernel arises in a network of coupled quantum harmonic oscillators. When the thermal Green’s function is restricted to the vacuum-plus-one-particle sector, it becomes, up to a multiplicative factor, a heat-kernel operator of the form $\exp (-\kappa \mathscr {L}_{\textrm{gen}})$, where *κ* depends on the inverse temperature and the oscillator parameters. Consequently, classical diffusion and thermal quantum propagation can generate the same observable relational geometry (Estrada 2026b). The geometry encoded by the response operator therefore provides the common object of study across these microscopic realizations.

### A single observation versus the complete response family

For a fixed initial condition $\textbf{c}_{0}$, the solution $\textbf{c}(t)$ describes one particular event of signal propagation. Such a trajectory may reveal which vertices are rapidly reached from a source, which regions evolve coherently, where bottlenecks occur, and how the system approaches equilibrium. Nevertheless, it displays only the relations expressed under the selected initial condition.

This distinction is essential. The operator $\mathscr {L}_{\textrm{gen}}$ determines a family of possible trajectories, whereas a particular $\textbf{c}_{0}$ selects only one member of that family. Changing the initial condition changes the observed propagation pattern while preserving the underlying substrate and its propagation law. GDT refers to the information supplied by one such realization as the *observational level*. This information describes a particular physical realization; integrating a sufficiently rich response family reveals the invariant relational organization encoded in the full response operator.

The Diffusion-Based Machine (DBM) provides an operational formulation of this distinction. A DBM encounters the relational substrate through the action of the propagation dynamics on distributed states. If it has access only to one realized trajectory, it can distinguish elements solely through the relations manifested in that realization. If it integrates a sufficiently rich family of responses, it can recover invariant relations that may remain invisible in every isolated trajectory.

In a neural system, ongoing sensory inputs, spontaneous activity, recurrent signaling, convergent projections, and temporal integration can continuously excite and combine different directions of the response space. The mathematical family of initial conditions represents this range of dynamically available perturbations.

### How propagation generates geometry

The passage from dynamics to geometry is particularly transparent for a connected undirected graph with a real symmetric Laplacian $\mathscr {L}$. Consider the propagation initiated independently from every vertex. If $\textbf{e}_{i}$ is the *i*th canonical basis vector, the response generated from vertex *i* at propagation time *τ* is

2.3 $$\begin{aligned} \textbf{c}^{(i)}(\tau )=e^{-\tau \mathscr {L}}\textbf{e}_{i}. \end{aligned}$$

Collecting these responses as columns gives

2.4 $$\begin{aligned} \mathscr {C}(\tau )=\left[ \textbf{c}^{(1)}(\tau )\;\cdots \;\textbf{c}^{(n)}(\tau )\right] =e^{-\tau \mathscr {L}}. \end{aligned}$$

Because $\mathscr {L}$ is symmetric,

2.5 $$\begin{aligned} \mathscr {C}(\tau )^{T}\mathscr {C}(\tau )=e^{-2\tau \mathscr {L}}. \end{aligned}$$

Introducing *t=2τ* and defining

2.6 $$\begin{aligned} \mathscr {X}(t)=e^{-t\mathscr {L}/2},\qquad \mathscr {K}(t)=e^{-t\mathscr {L}}, \end{aligned}$$

we obtain

2.7 $$\begin{aligned} \mathscr {X}(t)^{T}\mathscr {X}(t)=\mathscr {K}(t). \end{aligned}$$

The diffusion kernel $\mathscr {K}(t)$ is therefore a Gram matrix: a matrix of inner products between vectors, from which the pairwise Euclidean distances between those vectors can be read off directly. If

2.8 $$\begin{aligned} \mathscr {X}(t)=\left[ \textbf{x}_{1}(t)\;\cdots \;\textbf{x}_{n}(t)\right] , \end{aligned}$$

then

2.9 $$\begin{aligned} \textbf{x}_{i}(t)^{T}\textbf{x}_{j}(t)=\mathscr {K}_{ij}(t). \end{aligned}$$

Each system element is thus represented by a vector $\textbf{x}_{i}(t)$ in a Euclidean space, and the distance between two elements is

2.10 $$\begin{aligned} \mathscr {D}^{2}(t;i,j)=\left\| \textbf{x}_{i}(t)-\textbf{x}_{j}(t)\right\| ^{2}=\mathscr {K}_{ii}(t)+\mathscr {K}_{jj}(t)-2\mathscr {K}_{ij}(t) \end{aligned}$$

Ronald R Coifman et al. (2005); Coifman and Lafon (2006); Estrada (2012). This is the diffusion distance associated with the complete response family.

The squared norm of vertex *i* in this particular Gram realization is

$$\Vert x_{i}(t)\Vert ^{2}=\mathscr {K}_{ii}(t).$$

The norms depend on the diagonal entries of the heat kernel and can therefore vary across vertices. The circumcenter of the configuration can accordingly be displaced from the origin of the canonical Gram embedding $X(t)=e^{-tL/2}$. For the standard Laplacian of a connected undirected graph, the heat-kernel geometry is circum-Euclidean: the points admit a translated Euclidean realization on a common sphere. When all quantities $\mathscr {K}_{ii}(t)$ are equal, that sphere is centered at the origin of the Gram realization used above.

This geometry is intrinsic in the sense relevant to GDT. It is jointly determined by the physical relational substrate and by the dynamics supported by that substrate. Its construction follows directly from the physical response operator. Its distances, angles, symmetries, and equivalence classes describe how the elements participate in the entire space of possible propagation events.

A single trajectory supplies one vector-valued evolution, while the Gram matrix contains the pairwise inner products generated by the complete response family. Integrating multiple responses therefore does more than accumulate additional observations: it converts distributed dynamical information into a common relational domain.

### Propagation time and geometric differentiation

The parameter *t* is both a dynamical and a geometrical variable. At very short propagation times, from the truncation of the power series of the matrix exponential, we can get

2.11 $$\begin{aligned} \mathscr {K}(t)=e^{-t\mathscr {L}}\approx \mathscr {I}-t\mathscr {L}, \end{aligned}$$

where $\mathscr {I}$ is the identity matrix. So, the kernel is close to the identity and the response remains strongly localized. At very long times, conservative diffusion approaches equilibrium and progressively erases distinctions among vertices. The most informative geometry normally appears between these limiting regimes, after propagation has traveled sufficiently far to reveal mesoscopic and global organization but before equilibration has made the elements indistinguishable.

GDT therefore concerns the existence, differentiation, and temporal availability of a propagation-induced geometry. Its functional role depends on becoming available within the temporal window of neural processing.

### Comparing geometric and observational descriptions

The distinction between the complete diffusion geometry and a particular realized field can be quantified by comparing the classifications that they induce.

For each vertex *i*, define its geometric signature as the ordered list of its diffusion distances from all vertices,

2.12 $$\begin{aligned} \textbf{s}_{i}^{\textrm{geo}}(t)=\operatorname {sort}\left( \mathscr {D}(t;i,1),\ldots ,\mathscr {D}(t;i,n)\right) . \end{aligned}$$

Vertices with equal geometric signatures, within a specified numerical tolerance, are assigned to the same class. The resulting collection of classes defines the geometric partition $\Pi _{\textrm{geo}}(t)$.

Now consider one realized concentration field $\textbf{c}(t)$ generated from $\textbf{c}_{0}$. Its pairwise concentration differences are

2.13 $$\begin{aligned} \delta _{ij}^{\textrm{conc}}(t;\textbf{c}_{0})=\left| c_{i}(t)-c_{j}(t)\right| , \end{aligned}$$

and the corresponding concentration signature of vertex *i* is

2.14 $$\begin{aligned} \textbf{s}_{i}^{\textrm{conc}}(t;\textbf{c}_{0})=\operatorname {sort}\left( \delta _{i1}^{\textrm{conc}},\ldots ,\delta _{in}^{\textrm{conc}}\right) . \end{aligned}$$

These signatures define the concentration partition $\Pi _{\textrm{conc}}(t;\textbf{c}_{0})$. The crucial difference is that $\Pi _{\textrm{geo}}(t)$ depends on the complete response operator, whereas $\Pi _{\textrm{conc}}(t;\textbf{c}_{0})$ contains only the distinctions expressed in the selected realization.

Let $p_{a}(t)$ be the fraction of vertices in class *a* of the geometric partition, let $q_{b}(t;\textbf{c}_{0})$ be the fraction in class *b* of the concentration partition, and let $p_{ab}(t;\textbf{c}_{0})$ be their joint fraction. The corresponding partition entropies are

2.15 $$\begin{aligned} H_{\textrm{geo}}(t)=-\sum _{a}p_{a}(t)\log p_{a}(t) \end{aligned}$$

and

2.16 $$\begin{aligned} H_{\textrm{conc}}(t;\textbf{c}_{0})=-\sum _{b}q_{b}(t;\textbf{c}_{0})\log q_{b}(t;\textbf{c}_{0}). \end{aligned}$$

Their mutual information is

2.17 $$\begin{aligned} I(t;\textbf{c}_{0})=\sum _{a,b}p_{ab}(t;\textbf{c}_{0})\log \frac{p_{ab}(t;\textbf{c}_{0})}{p_{a}(t)q_{b}(t;\textbf{c}_{0})}. \end{aligned}$$

Provided that both entropies are nonzero, the normalized similarity between the partitions is

2.18 $$\begin{aligned} S_{\textrm{part}}(t;\textbf{c}_{0})=\frac{I(t;\textbf{c}_{0})}{\sqrt{H_{\textrm{geo}}(t)H_{\textrm{conc}}(t;\textbf{c}_{0})}}. \end{aligned}$$

The instantaneous partition gap is then

2.19 $$\begin{aligned} \Upsilon _{\textrm{part}}(t;\textbf{c}_{0})=1-S_{\textrm{part}}(t;\textbf{c}_{0}), \end{aligned}$$

and its temporal average over the interval [0, *T*] is

2.20 $$\begin{aligned} \overline{\Upsilon }_{\textrm{part}}(\textbf{c}_{0})=\frac{1}{T}\int _{0}^{T}\Upsilon _{\textrm{part}}(t;\textbf{c}_{0})\,dt. \end{aligned}$$

A partition gap close to zero indicates that the realized concentration field reproduces the classification induced by the complete geometry. A positive gap identifies additional relational distinctions available at the geometric level beyond those expressed by that particular realization.

The degree of differentiation within each description can be compared independently of the partition classes. Define the geometric signature differentiation by

2.21 $$\begin{aligned} I_{\textrm{sig}}(t)=\frac{1}{n^{2}}\sum _{i,j=1}^{n}\left\| \textbf{s}_{i}^{\textrm{geo}}(t)-\textbf{s}_{j}^{\textrm{geo}}(t)\right\| ^{2}, \end{aligned}$$

and its concentration counterpart by

2.22 $$\begin{aligned} I_{\textrm{sig}}^{\textrm{conc}}(t;\textbf{c}_{0})=\frac{1}{n^{2}}\sum _{i,j=1}^{n}\left\| \textbf{s}_{i}^{\textrm{conc}}(t;\textbf{c}_{0})-\textbf{s}_{j}^{\textrm{conc}}(t;\textbf{c}_{0})\right\| ^{2}. \end{aligned}$$

After normalizing both temporal profiles by their respective maxima over [0, *T*], their difference is

2.23 $$\begin{aligned} \widehat{I}_{\textrm{excess}}(t;\textbf{c}_{0})=\widehat{I}_{\textrm{sig}}(t)-\widehat{I}_{\textrm{sig}}^{\textrm{conc}}(t;\textbf{c}_{0}), \end{aligned}$$

with temporal mean

2.24 $$\begin{aligned} \frac{1}{T}\int _{0}^{T}\widehat{I}_{\textrm{excess}}(t;\textbf{c}_{0})\,dt. \end{aligned}$$

The two comparisons address complementary questions. The partition gap measures whether the observational and geometric levels classify the elements in the same way. The signature excess measures how strongly, and at what propagation times, those levels differentiate the elements. These operational measures test the distinction between information expressed in one realized field and information encoded by the complete propagation geometry.

### From diffusion geometry to the GDT hypothesis

The mathematical construction above establishes that structured propagation can generate an intrinsic relational geometry. GDT specifies the additional conditions under which that geometry could contribute to conscious experience.

The theory can be organized into six requirements.

1. A nontrivial relational substrate must be physically instantiated at a scale appropriate to the relevant dynamics.
2. The substrate must support distributed diffusion-like propagation and a sufficiently rich family of possible trajectories.
3. The response family must induce an intrinsic relational geometry, such as the kernel geometry constructed above.
4. The resulting geometry must be sufficiently differentiated and must emerge within a timescale accessible to the system.
5. Its geometric invariants must be intrinsically accessible. The relevant relations must be causally and functionally available inside the system.
6. The accessed invariants must be integrated and recursively used, allowing them to influence subsequent dynamics, functional organization, or structure.

These requirements describe the causal sequence

2.25 $$\begin{aligned} \begin{aligned}\text {relational structure}&\longrightarrow \text {propagation dynamics}\\&\longrightarrow \text {intrinsic geometry}\longrightarrow \text {intrinsic access}\\&\longrightarrow \text {recursive use and feedback}\\&\longrightarrow \text {modified structure and dynamics}. \end{aligned} \end{aligned}$$

The first four requirements specify a differentiated candidate geometry, termed proto-conscious within GDT to designate its role as a geometric prerequisite. According to GDT, conscious experience requires the additional intrinsic access to, and recursive causal use of, the invariant relations encoded by that geometry. The six requirements jointly distinguish the proposed organization of conscious systems from the broader class of systems supporting diffusion-induced geometry.

Within this framework, differentiation and unity are complementary properties. Differentiation arises because the elements occupy distinct relational positions and perform distinct roles in propagation. Unity arises because all of those positions are defined within a single metric configuration generated by common dynamics. GDT hypothesizes that qualitative experiences correspond to invariant organizations, regions, and transformations within an intrinsically accessed geometry. That correspondence is the theoretical proposal of GDT and the subject of its empirical predictions.

## Candidate color geometry: from colorness to particular contents

We now consider an illustrative construction showing how the Geometro-Diffusion Theory may be extended from the general conditions required for conscious experience to the organization of particular conscious contents. color provides a useful example because color experiences display both categorical and continuous structures. Red, green, blue, and yellow are commonly treated as distinguishable qualitative categories, but the colors within each category also vary continuously in hue, lightness, chroma, and similarity to other colors.

The present construction provides a mathematically controlled model of the relational organization of color content. color perception depends on retinal processing, chromatic adaptation, opponent mechanisms, cortical transformations, contextual effects, and individual variability (Maule et al. 2023). Within this biological setting, our example distinguishes three components:This distinction separates colorness, understood as the qualitative domain comprising chromatic qualia such as redness, blueness, greenness, and orangeness, from particular observations of color, such as a specific pale or vivid red, which constitute individual realizations within that domain.

- the diffusion-induced geometry that provides the relational capacity for color experience;
- the perceptual color domain within which particular color contents are organized;
- the contextual input that selects a particular region or position within that domain.

### A perceptually organized color domain

Here we consider CIELAB (International Organization for Standardization and International Commission on Illumination 2019) which provide a standardized and approximately perceptually uniform geometry suitable for constructing and testing the proposed distinction between modality-level colorness and particular color content.

In this framework, a color stimulus is represented by a vector

3.1 $$\begin{aligned} \textbf{p}=(L^{*},a^{*},b^{*})\in \mathcal {P}, \end{aligned}$$

where $L^{*}$ represents perceptual lightness, $a^{*}$ represents the red–green opponent direction, and $b^{*}$ represents the yellow–blue opponent direction. The domain $\mathcal {P}$ denotes the subset of CIELAB coordinates realizable under the adopted illuminant, reference white, display gamut, and viewing conditions.

It is often convenient to express the chromatic coordinates in cylindrical form. We define the CIELAB chroma by

3.2 $$\begin{aligned} C_{ab}^{*}=\sqrt{(a^{*})^{2}+(b^{*})^{2}}, \end{aligned}$$

and the hue angle by

3.3 $$\begin{aligned} h_{ab}=\operatorname {atan2}(b^{*},a^{*}). \end{aligned}$$

A color can then be represented as

3.4 $$\begin{aligned} \textbf{p}=(L^{*},C_{ab}^{*},h_{ab}). \end{aligned}$$

This representation separates three perceptually relevant transformations. A change in $L^{*}$ primarily modifies lightness, a change in $C_{ab}^{*}$ modifies chroma, and a change in $h_{ab}$ modifies hue.

The elementary colorimetric distance between two stimuli $\textbf{p}_{i}$ and $\textbf{p}_{j}$ is

3.5 $$\begin{aligned} \Delta E_{ab}(\textbf{p}_{i},\textbf{p}_{j})=\left[ (L_{i}^{*}-L_{j}^{*})^{2}+(a_{i}^{*}-a_{j}^{*})^{2}+(b_{i}^{*}-b_{j}^{*})^{2}\right] ^{1/2}. \end{aligned}$$

This Euclidean distance provides a standardized baseline for color comparisons. More refined color-difference formulae, such as CIEDE2000 (Sharma et al. 2005), will be used later on this work to correct known nonuniformities of CIELAB. Ultimately, however, the geometry relevant to subjective experience must be reconstructed from psychophysical judgments.

We therefore denote the subjective dissimilarity between two colors by $\delta _{\textrm{subj}}(\textbf{p}_{i},\textbf{p}_{j}).$ In a future empirical implementation, this quantity could be estimated from pairwise similarity or dissimilarity judgments. CIELAB consequently plays two restricted but important roles: it provides controlled coordinates for stimulus generation and defines a standard colorimetric geometry against which the subjective and neural geometries can be compared.

### Perceptual regions and color prototypes

The perceptual color domain can be divided into regions associated with color categories. Let $\mathcal {R}_{\alpha }\subseteq \mathcal {P}$ denote the region corresponding to a qualitative category *α*, such as red, green, blue, yellow, or orange. These regions are determined by the probability that an observer assigns a given stimulus to a particular color category.

For example, the red region can be defined as

3.6 $$\begin{aligned} \mathcal {R}_{\textrm{red}}=\left\{ \textbf{p}\in \mathcal {P}:P(\text {``red''}\mid \textbf{p})\ge \theta \right\} , \end{aligned}$$

where *θ* is a stated classification threshold. Analogous definitions apply to the other color regions. The boundaries between these regions may be graded and may depend on the observer, adaptation state, illumination, context, language, and experimental task.

Associated with every region $\mathcal {R}_{\alpha }$ is a representative point $\boldsymbol{\mu }_{\alpha }$, interpreted as the perceptual prototype of the corresponding category. If $\textbf{p}_{1},\ldots ,\textbf{p}_{m}$ are stimuli assigned to category *α*, the prototype can be defined as a Fréchet mean,

3.7 $$\begin{aligned} \boldsymbol{\mu }_{\alpha }=\underset{\textbf{q}\in \mathcal {R}_{\alpha }}{\operatorname {argmin}}\sum _{k=1}^{m}w_{k}\delta _{\textrm{subj}}^{2}(\textbf{q},\textbf{p}_{k}), \end{aligned}$$

where $w_{k}$ represents the empirical weight associated with stimulus $\textbf{p}_{k}$. In a preliminary computational construction, $\delta _{\textrm{subj}}$ may be replaced by $\Delta E_{ab}$ or another stated color-difference function. In the experimental version, both the regions and their prototypes should be estimated from behavioral data.

The distance of a stimulus $\textbf{p}$ from the red prototype,

3.8 $$\begin{aligned} \tau _{\textrm{red}}(\textbf{p})=\delta _{\textrm{subj}}\left( \textbf{p},\boldsymbol{\mu }_{\textrm{red}}\right) , \end{aligned}$$

provides a measure of its perceptual atypicality within the red region. Two stimuli may therefore belong to the same qualitative category while occupying different positions within it. This distinction makes it possible to represent pale, dark, vivid, desaturated, typical, and borderline reds as distinct positions within a common category.

### color observation as a diffusion consensus

We consider a connected graph *G=(V,E)* with *n* vertices and Laplacian $\mathscr {L}$. Each vertex carries a three-dimensional signal expressed relative to a neutral perceptual reference $\textbf{p}_{0}$. Let $\textbf{Z}^{0}\in \mathbb {R}^{n\times 3}$ denote the initial signal matrix. Its *i*th row,

3.9 $$\begin{aligned} \textbf{z}_{i}^{0}=\left( \Delta L_{i}^{*},\Delta a_{i}^{*},\Delta b_{i}^{*}\right) , \end{aligned}$$

represents a color-evidence signal relative to $\textbf{p}_{0}$ at vertex *i*. These variables are latent signal coordinates whose range can extend beyond the display gamut during propagation.

The three signal components diffuse independently according to

3.10 $$\begin{aligned} \frac{d\textbf{Z}(t)}{dt}=-\mathscr {L}\textbf{Z}(t),\qquad \textbf{Z}(0)=\textbf{Z}^{0}, \end{aligned}$$

with solution

3.11 $$\begin{aligned} \textbf{Z}(t)=e^{-t\mathscr {L}}\textbf{Z}^{0}. \end{aligned}$$

Because the graph is connected and the dynamics is conservative on the graph,

3.12 $$\begin{aligned} \lim _{t\rightarrow \infty }\textbf{Z}(t)=\textbf{1}\overline{\textbf{z}}, \end{aligned}$$

where

3.13 $$\begin{aligned} \overline{\textbf{z}}=\frac{1}{n}\textbf{1}^{T}\textbf{Z}^{0}. \end{aligned}$$

The consensus signal is decoded into the perceptual color descriptor

3.14 $$\begin{aligned} \overline{\textbf{p}}=\textbf{p}_{0}+\overline{\textbf{z}}. \end{aligned}$$

The initial conditions used in the calculations are selected so that $\overline{\textbf{p}}\in \mathcal {P}$. Thus, the decoded consensus corresponds to a physically realizable color, even though the intermediate signal amplitudes are interpreted as latent color evidence rather than literal displayed colors.

At the observational level, the DBM has access to the realized trajectory $\textbf{Z}(t)$ and to its consensus descriptor $\overline{\textbf{p}}$. It may consequently determine the color region containing the consensus, $\overline{\textbf{p}}\in \mathcal {R}_{\alpha },$ and measure its distance from the corresponding prototype,

3.15 $$\begin{aligned} \tau _{\alpha }(\overline{\textbf{p}})=\delta _{\textrm{subj}}\left( \overline{\textbf{p}},\boldsymbol{\mu }_{\alpha }\right) . \end{aligned}$$

As a pedagogical example we use here the six-vertex graph formed by two triangles connected by the edge joining vertices 3 and 4. The signal is initialized at vertex 1. We select two controlled red stimuli with the same lightness and hue angle, $L^{*}=60$, $h_{ab}=25^{\circ }$ but with different chroma. Along this constant-lightness and constant-hue ray, the largest value of $C_{ab}^{*}$ at the specified lightness and hue for which conversion from CIELAB, using a D65 reference white (see International Organization for Standardization and International Commission on Illumination 2019), produces an in-gamut sRGB color, namely, one whose three encoded channel values remain within the interval [0, 1], is $C_{\max }^{*}\approx 72.6791.$

To avoid stimuli lying directly on the gamut boundary, the lower- and higher-chroma stimuli are selected at *40%* and *80%*, respectively, of this maximum. Their cylindrical CIELAB coordinates are

3.16 $$\begin{aligned} \overline{\textbf{p}}_{A}=(60,29.0716,25^{\circ }),\qquad \overline{\textbf{p}}_{B}=(60,58.1433,25^{\circ }), \end{aligned}$$

or, equivalently, in Cartesian CIELAB coordinates,

3.17 $$\begin{aligned} \overline{\textbf{p}}_{A}=(60,26.3478,12.2862),\qquad \overline{\textbf{p}}_{B}=(60,52.6957,24.5724). \end{aligned}$$

Their corresponding sRGB coordinates, used only to render the figures, are approximately

3.18 $$\begin{aligned} \textbf{r}_{A}=(0.7652,0.4959,0.4871),\qquad \textbf{r}_{B}=(0.9265,0.3946,0.4094). \end{aligned}$$

The two stimuli have equal lightness and hue and differ in chroma. Their Euclidean CIELAB separation and CIEDE2000 color difference are, respectively

3.19 $$\begin{aligned} \Delta E_{ab}\left( \overline{\textbf{p}}_{A},\overline{\textbf{p}}_{B}\right) \approx 29.0716,\qquad \Delta E_{00}\left( \overline{\textbf{p}}_{A},\overline{\textbf{p}}_{B}\right) \approx 9.8274. \end{aligned}$$

For reference, an illustrative red prototype is placed at *65%* of the maximum chroma on the same constant-lightness and constant-hue ray,

3.20 $$\begin{aligned} \boldsymbol{\mu }_{\textrm{red}}^{(0)}=(60,47.2414,25^{\circ })=(60,42.8153,19.9651). \end{aligned}$$

This point provides a computational reference for the example. Its CIEDE2000 distances from the lower- and higher-chroma stimuli are approximately 6.7124 and 3.2349, respectively. In an empirical implementation, the prototype would instead be estimated from behavioural categorization and similarity data.

To implement the two initial conditions, let

3.21 $$\begin{aligned} \textbf{a}(0)=6\textbf{e}_{1},\qquad \textbf{a}(t)=e^{-t\mathscr {L}}\textbf{a}(0). \end{aligned}$$

Because $\textbf{1}^{T}\textbf{a}(t)=6$, the mean amplitude is equal to one at every time. For either target $\overline{\textbf{p}}_{\alpha }$, where $\alpha \in \{A,B\}$, the latent color-evidence field is

3.22 $$\begin{aligned} \textbf{Z}_{\alpha }(t)=\textbf{a}(t)\left( \overline{\textbf{p}}_{\alpha }-\textbf{p}_{0}\right) ,\qquad \textbf{p}_{0}=(60,0,0). \end{aligned}$$

Consequently,

3.23 $$\begin{aligned} \frac{1}{6}\textbf{1}^{T}\textbf{Z}_{\alpha }(t)=\overline{\textbf{p}}_{\alpha }-\textbf{p}_{0}, \end{aligned}$$

and the decoded consensus is exactly $\overline{\textbf{p}}_{\alpha }$. The amplified source signal represents latent opponent-color evidence. For visualization, the local amplitude is mapped smoothly into a bounded chroma interval by

3.24 $$\begin{aligned} \omega (a)=1+0.22\tanh \left( \frac{a-1}{0.40}\right) , \end{aligned}$$

and the displayed descriptor at vertex *i* is

3.25 $$\begin{aligned} \textbf{p}_{i,\alpha }^{\textrm{disp}}(t)=\textbf{p}_{0}+\omega \left( a_{i}(t)\right) \left( \overline{\textbf{p}}_{\alpha }-\textbf{p}_{0}\right) . \end{aligned}$$

This visualization map satisfies *ω (1)=1*, preserves differences on both sides of consensus, and keeps all displayed colors inside the selected sRGB gamut.

**Fig. 2:**
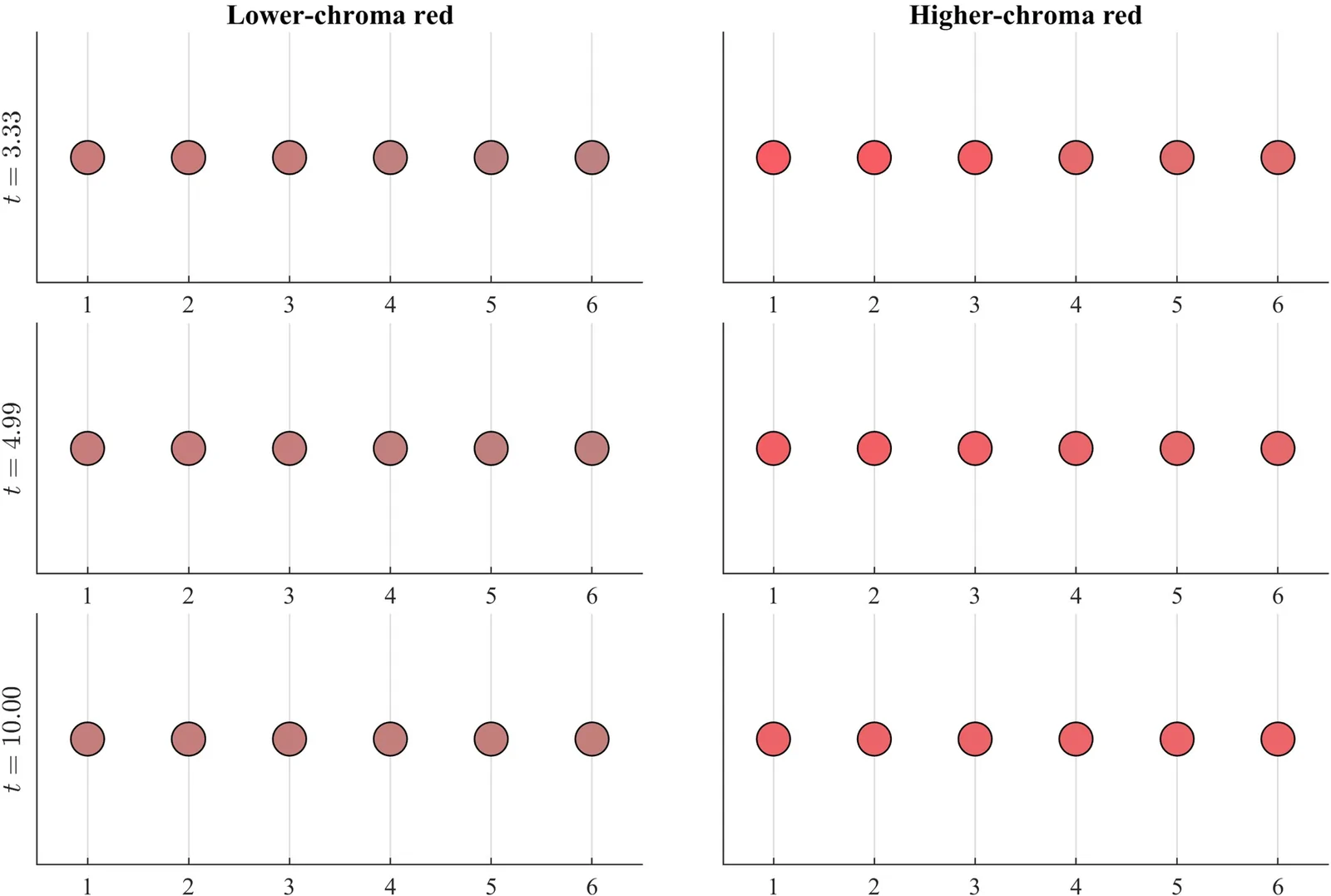
Diffusion of two controlled CIELAB red signals on the six-vertex graph formed by two triangles connected through the edge joining vertices 3 and 4. The rows show the vertex states at *t=3.33*, 4.99, and 10. The left and right columns have the same lightness, $L^{*}=60$, and hue angle, $h_{ab}=25^{\circ }$, but different chroma: $C_{ab}^{*}=29.0716$ for the lower-chroma red and $C_{ab}^{*}=58.1433$ for the higher-chroma red. The latent signal is initialized at vertex 1 and propagates according to standard diffusion. The colors shown at the vertices are gamut-valid visualizations obtained by mapping the latent opponent-color evidence into a bounded chroma interval. Both processes approach a spatially uniform consensus while retaining their different positions along the common red-hue direction

Figure 2 shows the resulting evolution at *t=3.33*, 4.99, and 10. At the first time, the two triangles retain visibly different color-evidence levels because the bridge between vertices 3 and 4 delays equilibration. The difference is smaller at *t=4.99*, and by *t=10* the six vertices are close to their common consensus. The spatial propagation pattern is the same in the two columns because the graph, Laplacian, source vertex, and diffusion times are identical. Only the chroma of the decoded content changes.

We call this the *observational level*. At this level, the DBM can report the detection of a particular red stimulus and distinguish

it from other red stimuli. It therefore corresponds to a report of the form: “I see a particular shade of red.” The realized trajectory describes the observed color-related signal. The complete response family specifies the invariant geometric organization within which this observation is embedded.

### The diffusion-induced scaffold of colorness

We now consider the geometry generated by the complete family of diffusion responses. Let

3.26 $$\begin{aligned} \mathscr {L}=\mathcal {U}\boldsymbol{\Lambda }\mathcal {U}^{T} \end{aligned}$$

be the spectral decomposition of the graph Laplacian, and define

3.27 $$\begin{aligned} \textbf{x}_{v}(t)=e^{-t\boldsymbol{\Lambda }/2}\mathcal {U}^{T}\textbf{e}_{v}. \end{aligned}$$

The collection $\mathcal {S}_{t}=\left\{ \textbf{x}_{v}(t):v\in V\right\} \subset \mathbb {R}^{n}$ forms the diffusion-induced geometric scaffold. Its pairwise Euclidean distances coincide with the diffusion distances, $\left\| \textbf{x}_{i}(t)-\textbf{x}_{j}(t)\right\| ^{2}.$

The full-dimensional vectors can have different norms about the origin for a general graph. In the present non-vertex-transitive example, at the geometric time *t=0.35* their norms separate into two symmetry classes. Vertices 1, 2, 5, and 6 have norm approximately 0.7503, whereas bridge vertices 3 and 4 have norm approximately 0.6552. The complete diffusion-induced relational scaffold captures these differences in position. A common spherical envelope provides a visual aid for displaying the scaffold.

In the present illustrative construction, the invariant organization of $\mathcal {S}_{t}$ is associated with *colorness*: the general capacity of the system to organize color-like qualitative contents within an integrated relational domain. This identification is a theoretical hypothesis within GDT. According to the six requirements introduced previously, the geometry must also be sufficiently differentiated, arise on a relevant timescale, be intrinsically accessible, and participate in recursive causal interactions. The present section specifies the geometric component required for the subsequent construction.

An important feature of $\mathcal {S}_{t}$ is that it is determined by the substrate and its propagation operator, independently of the particular color-evidence matrix $\textbf{Z}^{0}$. Different color inputs can therefore generate different observational trajectories and consensus descriptors while sharing the same underlying diffusion geometry. This is the basis for distinguishing the modality-level capacity for colorness from the particular color content instantiated on a given occasion.

The diffusion geometry $\mathcal {S}_{t}$ therefore represents the modality-level scaffold. As developed in the next section, redness appears when this scaffold is combined with the geometry of the instantiated color content, giving rise to a product geometry of color experience in which redness occupies a particular region.

### The product geometry of color experience

Let $\mathcal {P}$ denote the perceptual color domain and let $\mathcal {S}_{t}$ denote the diffusion-induced scaffold. We define the extended scaffold–content domain as $\mathcal {E}_{t}=\mathcal {S}_{t}\times \mathcal {P}.$ Its individual elements are pairs $(\textbf{x}_{v}(t),\textbf{p})\in \mathcal {S}_{t}\times \mathcal {P},$ where $\textbf{x}_{v}(t)$ is the diffusion coordinate of vertex *v* and $\textbf{p}$ is a perceptual coordinate. For a fixed decoded color descriptor $\textbf{p}\in \mathcal {P}$, the corresponding fiber is $\mathcal {E}_{t}(\textbf{p})=\mathcal {S}_{t}\times \{\textbf{p}\}.$ To refer to this fiber together with the content that identifies it, we introduce the structured object $\mathfrak {E}_{t}(\textbf{p})=\left( \mathcal {S}_{t},\textbf{p}\right) .$ The notation $\mathfrak {E}_{t}(\textbf{p})$ describes the complete scaffold carrying the particular content $\textbf{p}$, and thus identifies the corresponding fiber of $\mathcal {S}_{t}\times \mathcal {P}$. The two components have different roles: $\mathcal {S}_{t}$ represents the modality-level relational scaffold associated with colorness, whereas $\textbf{p}$ specifies the particular position occupied within the perceptual color domain. Thus,Two experiential fibers represented by $\mathfrak {E}_{t}(\textbf{p}_{A})=\left( \mathcal {S}_{t},\textbf{p}_{A}\right)$ and $\mathfrak {E}_{t}(\textbf{p}_{B})=\left( \mathcal {S}_{t},\textbf{p}_{B}\right)$ share the same modality-level scaffold but differ in their particular contents. If $\textbf{p}_{A},\textbf{p}_{B}\in \mathcal {R}_{\textrm{red}},$ both correspond to forms of redness, although they may differ in lightness, chroma, hue, or typicality. If instead $\textbf{p}_{A}\in \mathcal {R}_{\textrm{red}}$ and $\textbf{p}_{B}\in \mathcal {R}_{\textrm{blue}},$ the same scaffold supports contents belonging to two different color categories.

- $\mathcal {S}_{t}$ represents the modality-level geometric scaffold;
- $\textbf{p}\in \mathcal {R}_{\textrm{red}}$ specifies an experience belonging to the red region;
- the position of $\textbf{p}$ within $\mathcal {R}_{\textrm{red}}$ distinguishes different kinds of red;
- the distance from $\textbf{p}$ to $\boldsymbol{\mu }_{\textrm{red}}$ quantifies its typicality relative to the red prototype.

One admissible metric on the product domain is

3.28 $$\begin{aligned} d_{\mathcal {E}}^{2}\left( (\textbf{x}_{i},\textbf{p}_{A}),(\textbf{x}_{j},\textbf{p}_{B})\right) =\mathscr {D}^{2}(t;i,j)+\lambda ^{2}\delta _{\mathcal {P}}^{2}\left( \textbf{p}_{A},\textbf{p}_{B}\right) , \end{aligned}$$

where *λ>0* fixes the relative scale of the scaffold and content components, and $\delta _{\mathcal {P}}$ is the adopted distance on the perceptual color domain. This expression defines a standard weighted product metric whenever $\mathscr {D}$ and $\delta _{\mathcal {P}}$

are metrics. We use $\Delta E_{ab}$ for this product-metric construction. CIEDE2000 serves as a reference dissimilarity in the comparisons below, while the product metric uses $\Delta E_{ab}$. In a psychophysical implementation, $\delta _{\mathcal {P}}$ can instead be a metric reconstructed from subjective judgments.

This product representation is preferable to assigning every member of a color category to a single prototype. Mapping all red stimuli onto $\boldsymbol{\mu }_{\textrm{red}}$ would preserve categorical membership but eliminate the internal geometry of the red region. By retaining the continuous coordinate $\textbf{p}$, the model represents both qualitative category and within-category variation.

**Fig. 3:**
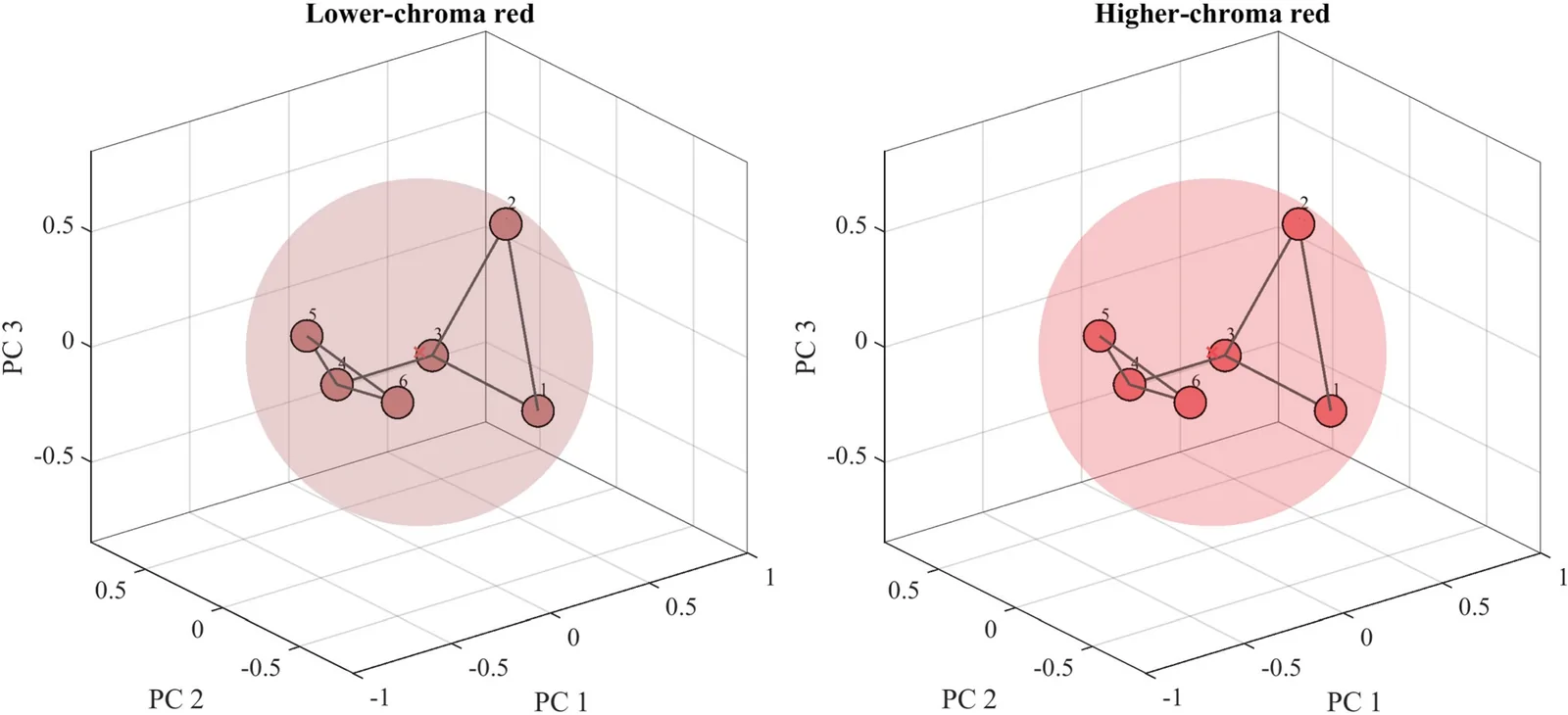
Two fibers of the same diffusion-induced scaffold corresponding to different positions within the red region of the CIELAB domain. The six-dimensional diffusion coordinates of the graph are calculated at *t=0.35* and projected onto the first three principal components for visualization. The geometry is identical in both panels: all vertex positions, edges, the common center, the spherical visual envelope, the axis limits, and the viewing direction are unchanged. The left fiber carries the lower-chroma content $\overline{\textbf{p}}_{A}=(60,29.0716,25^{\circ })$ in cylindrical CIELAB coordinates, whereas the right fiber carries the higher-chroma content $\overline{\textbf{p}}_{B}=(60,58.1433,25^{\circ })$. Thus, the modality-level scaffold associated with colorness is preserved while the particular red content changes. The translucent sphere provides an illustrative envelope of the common scaffold

Figure 3 illustrates this product construction for the two calculated red contents. The six-dimensional diffusion coordinates at *t=0.35* are projected onto their first three principal components for visualization. Exactly the same projected vertex positions, graph edges, center, spherical envelope, axis limits, and viewing angle are used in both panels. The only difference is the CIELAB content associated with the scaffold. The translucent sphere provides a common visual envelope representing the modality-level capacity for colorness. The full diffusion coordinates determine the distances, and their three-dimensional PCA projections can have different norms about the origin.

Within the interpretation proposed by GDT, we call this the candidate experiential level. The present construction specifies its relational content. Within GDT, intrinsic access and recursive use connect this content to experience: at this level, the DBM accesses the position of a particular red within the product geometry of color experience. In the simplified reporting language used here, this corresponds to the statement: “I experience the redness of this red.”

### Observation, colorness, and particular content

The construction distinguishes the realized trajectory and its decoded consensus, the input-independent scaffold $\mathcal {S}_{t}$, and the particular content carried on that scaffold. The structured fiber $\mathfrak {E}_{t}(\overline{\textbf{p}})=(\mathcal {S}_{t},\overline{\textbf{p}})$ specifies a color within the common modality-level organization. Colorness refers to that shared relational capacity; redness identifies a region $\mathcal {R}_{\textrm{red}}$ of the content domain; and a particular shade occupies a position within that region. Within GDT, intrinsic access and recursive causal use connect this structured content to experience.

### From a colorimetric construction to an empirical geometry

The preceding construction uses CIELAB (International Organization for Standardization and International Commission on Illumination 2019) as an approximately perceptually organized reference space. An empirical reconstruction from subjective judgments provides the corresponding geometry of experienced color relations.

Consider a controlled set of color stimuli

3.29 $$\begin{aligned} \mathcal {Q}=\left\{ \textbf{p}_{1},\ldots ,\textbf{p}_{m}\right\} \subset \mathcal {P}. \end{aligned}$$

Pairwise psychophysical judgments define the subjective dissimilarity matrix

3.30 $$\begin{aligned} \textbf{D}^{\textrm{subj}}=\left[ \delta _{\textrm{subj}}(\textbf{p}_{i},\textbf{p}_{j})\right] _{i,j=1}^{m}. \end{aligned}$$

Multidimensional scaling or a related embedding procedure can then be used to obtain subjective coordinates $\textbf{y}_{1},\ldots ,\textbf{y}_{m}\in \mathbb {R}^{q}$ such that

3.31 $$\begin{aligned} \left\| \textbf{y}_{i}-\textbf{y}_{j}\right\| \approx \delta _{\textrm{subj}}(\textbf{p}_{i},\textbf{p}_{j}). \end{aligned}$$

These coordinates define an empirical perceptual domain $\mathcal {P}_{\textrm{subj}}$. Its dimensionality, curvature, anisotropies, category boundaries, and local deformations become measurable quantities.

The neural data associated with the same stimuli can likewise be used to construct different representational geometries. Let $\textbf{D}^{\textrm{act}}$ denote a distance matrix derived from neural activation patterns, and let $\textbf{D}^{\textrm{diff}}$ denote the distance matrix generated by the estimated neural propagation operator. The corresponding CIELAB distance matrix is

3.32 $$\begin{aligned} \textbf{D}^{\textrm{CIELAB}}=\left[ \Delta E_{ab}(\textbf{p}_{i},\textbf{p}_{j})\right] _{i,j=1}^{m}. \end{aligned}$$

The central empirical question is then whether the subjective geometry is better predicted by the diffusion-induced neural geometry than by the physical stimulus geometry or by differences between raw neural activation patterns. In its strongest form, the prediction is

3.33 $$\begin{aligned} \textbf{D}^{\textrm{diff}}\text { explains variability in }\textbf{D}^{\textrm{subj}}\text { beyond that explained by }\textbf{D}^{\textrm{CIELAB}}\text { and }\textbf{D}^{\textrm{act}}. \end{aligned}$$

The present calculations establish the controlled colorimetric construction and demonstrate how distinct CIELAB (International Organization for Standardization and International Commission on Illumination 2019) contents can be combined with an unchanged diffusion-induced scaffold. The later sections extend this construction computationally to neuronal dynamics and a human structural connectome. A direct empirical test of the stronger prediction will require future reconstruction of psychophysical similarity spaces and neural representational and propagation geometries, followed by quantitative comparison of their distance matrices, which are obviously out of the scope of the present work.

For the present theoretical construction, the essential conclusion is that color content should be represented as a continuous position within an empirically constrained perceptual domain. The diffusion-induced scaffold supplies the modality-level relational organization, while the position within the perceptual domain specifies the particular content instantiated on that scaffold. In this way, GDT distinguishes the capacity for colorness, the categorical quality of redness, and the particular character of an individual red experience.

## Diffusion-induced scaffolds with dynamical decorations

The construction developed in the preceding section distinguishes the diffusion-induced scaffold $\mathcal {S}_{t}$ from the perceptual coordinate $\textbf{p}\in \mathcal {P}$ that specifies a particular color content. A further distinction is required when the vertices of the underlying network possess their own internal dynamics. Neurons, neuronal populations, and cortical regions each support time-dependent activity that may vary across vertices even when their relational positions in the diffusion geometry are equivalent.

This motivates the introduction of a *dynamically decorated diffusion geometry*. The diffusion-induced scaffold describes the invariant relational organization generated by propagation, whereas the decoration records the instantaneous internal state carried by each embedded vertex. The two components have complementary roles. The colors used below visualize neuronal activity through a plotting colormap, while the CIELAB coordinates in the preceding section represent color content.

### Two timescales: internal dynamics and network propagation

We consider again the graph *G=(V,E)* formed by two triangles connected by a single edge. Let *n=|V|* and let $\mathscr {L}$ be its combinatorial Laplacian. Two temporal variables are introduced:

- *τ* denotes the internal neuronal time along which each vertex generates its local activity;
- *t* denotes the propagation or integration time along which a fixed internal activity pattern is distributed across the network.

This is a two-time construction. For every fixed value of *τ*, the vector

4.1 $$\begin{aligned} \textbf{c}(0,\tau )=\left( c_{1}(0,\tau ),\ldots ,c_{n}(0,\tau )\right) ^{T} \end{aligned}$$

provides an initial condition for the propagation dynamics in *t*. The functions $c_{v}(0,\tau )$ may be generated by an Izhikevich model (Izhikevich 2003, 2007), a FitzHugh–Nagumo model (Cebrián-Lacasa et al. 2024), or another neurodynamical system. Thus, varying *τ* changes the internal activity pattern supplied to the network, whereas varying *t* reveals how that particular pattern is integrated by the relational substrate. Hereafter we will use the Izhikevich dynamics, which provide a biologically plausible neuronal realization of the internal dynamical decoration. The construction is intentionally modality-independent and can therefore be applied to color-related or other neuronal systems.

We use the semilinear diffusion equation

4.2 $$\begin{aligned} \frac{\partial \textbf{c}}{\partial t}(t,\tau )=-\mathscr {L}\textbf{c}(t,\tau )+\phi \left( \frac{1}{n}\textbf{1}^{T}\textbf{c}(t,\tau )\right) \textbf{1}, \end{aligned}$$

with

4.3 $$\begin{aligned} \textbf{c}(0,\tau )=\textbf{a}(\tau ), \end{aligned}$$

where $\textbf{a}(\tau )$ is the vector of internally generated neuronal activities. The scalar function *ϕ* acts only on the consensus component. It may represent a global nonlinear modulation, production term, or common drive whose value depends on the instantaneous network mean.

In the calculations below, we use

4.4 $$\begin{aligned} \phi (q)=\alpha q-\beta q^{3},\qquad \alpha =1,\qquad \beta =0.45. \end{aligned}$$

The internal activity is generated with regular-spiking Izhikevich neurons. Small deterministic variations in their parameters and initial conditions produce distinct signals at the six vertices. Each signal is centered and normalized to unit standard deviation before it is used as an initial condition for network propagation. This normalization preserves the temporal organization of the spikes while placing the six neuronal signals on comparable numerical scales.

Qualitatively, the neuronal signals provide the time-dependent *decoration* initially carried by the vertices, whereas the graph Laplacian determines how differences among those signals propagate through the network. For each fixed internal time *τ*, diffusion progressively attenuates the disagreement components of the vertex activities and thereby drives the network towards synchronization. This synchronization changes the activity displayed on the vertices while preserving the Laplacian, heat kernel, and diffusion geometry. The model thus describes neuronal decoration evolving on a prescribed diffusion scaffold. A state-dependent propagation operator would extend this construction to include feedback from the decoration to the scaffold.

The variable *t* is an operational propagation scale used to examine how activity available at internal time *τ* is reorganized by the network. In a biological implementation, the two variables may correspond to processes occurring simultaneously but at distinguishable characteristic timescales.

### Consensus and disagreement components

Define the consensus projector

4.5 $$\begin{aligned} \mathscr {J}=\frac{1}{n}\textbf{1}\textbf{1}^{T}, \end{aligned}$$

and the complementary disagreement projector

4.6 $$\begin{aligned} \mathscr {P}=\mathscr {I}-\mathscr {J}. \end{aligned}$$

Every state decomposes uniquely as

4.7 $$\begin{aligned} \textbf{c}(t,\tau )=\overline{c}(t,\tau )\textbf{1}+\textbf{v}(t,\tau ), \end{aligned}$$

where

4.8 $$\begin{aligned} \overline{c}(t,\tau )=\frac{1}{n}\textbf{1}^{T}\textbf{c}(t,\tau ),\qquad \textbf{v}(t,\tau )=\mathscr {P}\textbf{c}(t,\tau ). \end{aligned}$$

The disagreement vector satisfies

4.9 $$\begin{aligned} \textbf{1}^{T}\textbf{v}(t,\tau )=0. \end{aligned}$$

Since $\mathscr {L}\textbf{1}=\textbf{0}$, $\mathscr {P}\mathscr {L}=\mathscr {L}\mathscr {P}=\mathscr {L}$, and $\mathscr {P}\textbf{1}=\textbf{0}$, application of $\mathscr {P}$ to the semilinear equation gives

4.10 $$\begin{aligned} \frac{\partial \textbf{v}}{\partial t}(t,\tau )=-\mathscr {L}\textbf{v}(t,\tau ). \end{aligned}$$

Consequently,

4.11 $$\begin{aligned} \textbf{v}(t,\tau )=e^{-t\mathscr {L}}\textbf{v}(0,\tau ). \end{aligned}$$

The nonlinear term therefore changes only the consensus component. Indeed,

4.12 $$\begin{aligned} \frac{\partial \overline{c}}{\partial t}(t,\tau )=\phi \left( \overline{c}(t,\tau )\right) . \end{aligned}$$

For the cubic function used here, the consensus equation can be solved explicitly. If $q_{0}=\overline{c}(0,\tau )$, then

4.13 $$\begin{aligned} \overline{c}(t,\tau )=\operatorname {sgn}(q_{0})\left[ \frac{\alpha q_{0}^{2}}{\beta q_{0}^{2}+\left( \alpha -\beta q_{0}^{2}\right) e^{-2\alpha t}}\right] ^{1/2}, \end{aligned}$$

with $\overline{c}(t,\tau )=0$ when $q_{0}=0$. This expression was used directly in the numerical implementation, avoiding the need to solve a separate scalar differential equation for every sampled value of *τ*.

For a connected graph, the nonzero Laplacian eigenvalues are positive and

4.14 $$\begin{aligned} \lim _{t\rightarrow \infty }\textbf{v}(t,\tau )=\textbf{0}. \end{aligned}$$

Thus, for every fixed internal time *τ*, propagation progressively removes differences among vertices, while the common mode evolves according to the scalar nonlinear equation.

A normalized disagreement index can be defined by

4.15 $$\begin{aligned} \mathcal {E}_{\textrm{dis}}(t,\tau )=\frac{\left\| \mathscr {P}\textbf{c}(t,\tau )\right\| ^{2}}{\left\| \mathscr {P}\textbf{c}(0,\tau )\right\| ^{2}}, \end{aligned}$$

whenever the denominator is nonzero. It satisfies

4.16 $$\begin{aligned} \mathcal {E}_{\textrm{dis}}(0,\tau )=1,\qquad \lim _{t\rightarrow \infty }\mathcal {E}_{\textrm{dis}}(t,\tau )=0. \end{aligned}$$

The calculation of $\mathcal {E}_{\textrm{dis}}(t,\tau )$ provides a quantitative complement to the visual progression from heterogeneous activity to synchronization. We also calculate its aggregate version over the sampled internal-time interval,

4.17 $$\begin{aligned} \overline{\mathcal {E}}_{\textrm{dis}}(t)=\frac{\left\| \mathscr {P}\textbf{C}(t)\right\| _{F}^{2}}{\left\| \mathscr {P}\textbf{C}(0)\right\| _{F}^{2}}, \end{aligned}$$

where the columns of $\textbf{C}(t)$ are the states associated with the sampled values of *τ* and $\Vert \cdot \Vert _{F}$ is the Frobenius norm.

### Why the diffusion scaffold is preserved

The diffusion-induced scaffold is obtained from the propagation operator $\mathscr {K}(t)$ through the coordinates $\textbf{x}_{v}(t)$. The corresponding geometric object is $\mathcal {S}_{t}=\left\{ \textbf{x}_{v}(t):v\in V\right\} .$ Its pairwise distances are $\mathscr {D}^{2}(t;i,j)$. The semilinear consensus term preserves the disagreement propagator $e^{-t\mathscr {L}}$. Equivalently, it adds a common component parallel to $\textbf{1}$ and therefore disappears when differences between vertex responses are considered. For the equal-mass family of vertex-source responses used to reconstruct $\mathcal {S}_{t}$, the nonlinear consensus contribution is the same for every source and cancels from all pairwise response differences. The diffusion distances and the resulting scaffold are therefore identical to those obtained from standard linear diffusion.

This invariance has a precise scope. It follows because the nonlinear term is spatially uniform and acts exclusively in the consensus direction. A nonlinear term depending separately on the vertex states, or a state-dependent propagation operator, could modify the disagreement dynamics and hence deform the induced geometry. The present model isolates the case in which internal or global nonlinear activity changes the state carried by a preserved scaffold.

The consensus-directed form of the nonlinearity establishes this invariance algebraically. The numerical comparison below checks this exact algebraic result and its implementation. For the six canonical vertex sources, all of which have initial mean 1/6, the semilinear response matrix is

4.18 $$\begin{aligned} \mathscr {R}_{\textrm{semi}}(t)=e^{-t\mathscr {L}}+\left[ \overline{c}(t)-\frac{1}{6}\right] \textbf{1}\textbf{1}^{T}. \end{aligned}$$

The second term is common to every response column and therefore cancels from every pairwise column difference. Across the complete propagation-time grid, the maximum numerical difference between the linear and semilinear distance matrices remained below $10^{-10}$, confirming equality up to numerical roundoff.

### The observational dynamics

**Fig. 4:**
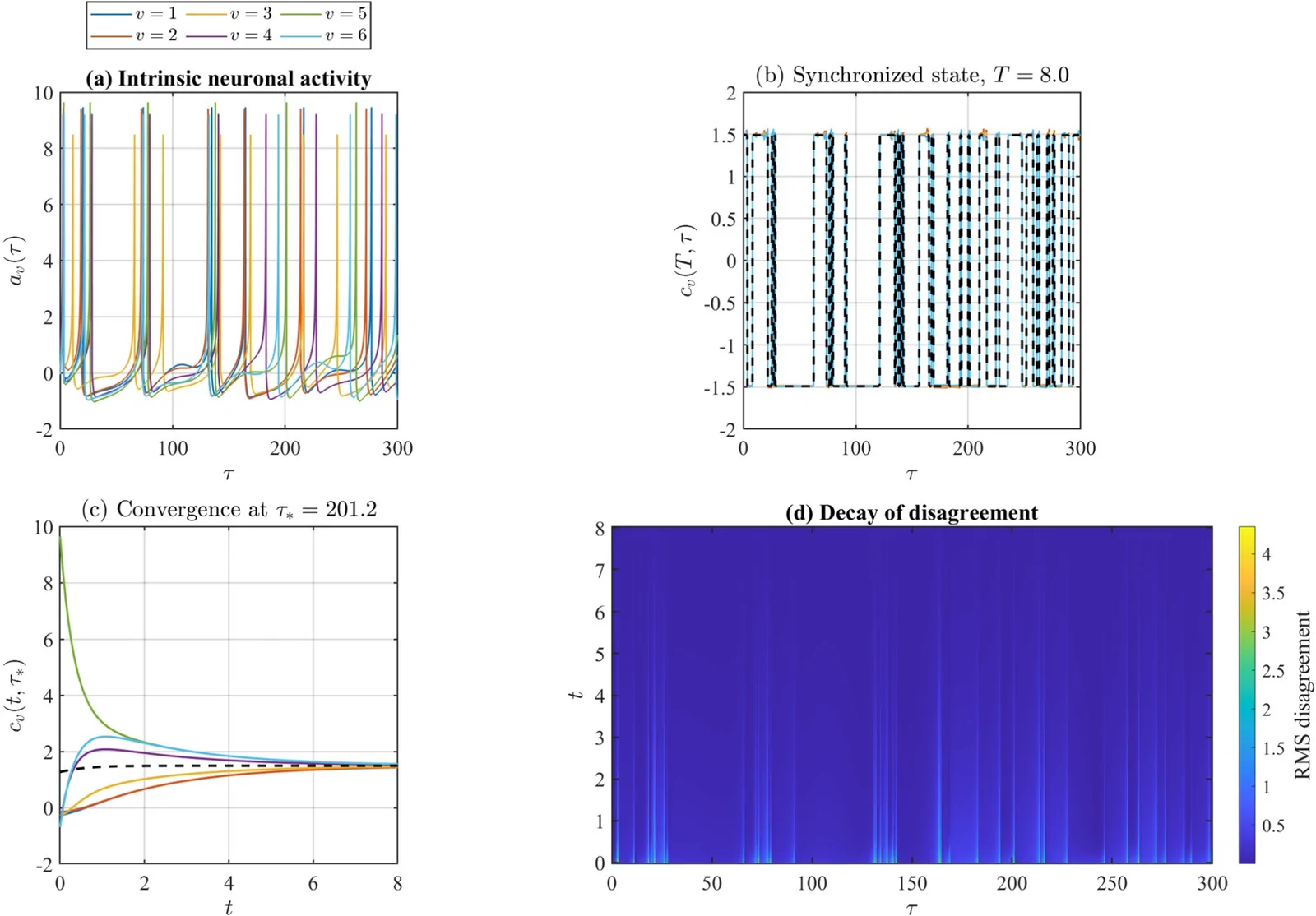
Observational analysis of internally generated neuronal activity and its semilinear propagation on the six-vertex graph. (a) Normalized intrinsic Izhikevich activity $a_{v}(\tau )$ generated at the six vertices before network integration. (b) Vertex signals at the final propagation time *T=8*; the almost complete overlap demonstrates spatial synchronization, and the dashed curve is the nonlinear consensus mode. (c) Convergence of the six vertex states toward the consensus trajectory at the automatically selected internal time $\tau _{*}$ shown in the panel. (d) Root-mean-square disagreement across the complete *(t,τ )* domain. The parula scale in panel (d) represents disagreement magnitude and is unrelated to the CIELAB perceptual coordinates introduced in Sect. 3

At the observational level, the DBM has access to the realized neuronal activity and its propagation. Integrating the response family provides access to the complete geometry. Figure 4 uses internally generated Izhikevich activity at the six vertices and displays four complementary descriptions:

- the six intrinsic neuronal signals $a_{v}(\tau )$ before network integration;
- the six nearly coincident signals at the final propagation time *T=8*, together with the nonlinear consensus mode;
- the convergence of the six vertex states as *t* increases at an automatically selected internal time $\tau _{*}$;
- the root-mean-square disagreement over the complete *(t,τ )* domain.

These panels distinguish local temporal complexity from network-level integration. The first panel describes what is generated inside the vertices. Panel (b) shows that propagation produces an almost spatially uniform waveform by *T=8*. Panel (c) isolates the convergence process at the value $\tau _{*}$ for which the initial cross-vertex variance is maximal within the interval $40\le \tau \le 280$. Panel (d) demonstrates that disagreement decreases throughout the sampled internal-time domain as *t* increases.

### Dynamically decorated diffusion geometries

At every pair *(t,τ )*, the internal state assigns a scalar value $c_{v}(t,\tau )$ to the embedded vertex $\textbf{x}_{v}(t)\in \mathcal {S}_{t}$. We therefore define the dynamically decorated scaffold by $\mathfrak {D}_{t,\tau }=\left( \mathcal {S}_{t},c(\cdot ,t,\tau )\right) .$ The first component specifies the relational positions of the vertices, while the second specifies the activity attached to those positions. A plotting colormap displays the neuronal values $c_{v}(t,\tau )$ attached to the scaffold.

When a particular conscious content $\textbf{p}\in \mathcal {P}$ is also included, the complete object becomes $\mathfrak {E}_{t,\tau }(\textbf{p})=\left( \mathcal {S}_{t},\textbf{p},c(\cdot ,t,\tau )\right) .$ The three entries play different roles:Section 3 considered two different values of $\textbf{p}$ attached to the same $\mathcal {S}_{t}$. The present construction considers a time-dependent decoration $c(\cdot ,t,\tau )$ on that scaffold. In a more complete model, both the perceptual coordinate and the activity decoration may vary, but they remain conceptually and mathematically distinguishable.

- $\mathcal {S}_{t}$ is the modality-level relational scaffold generated by propagation;
- $\textbf{p}$ specifies the particular perceptual content within the appropriate perceptual domain;
- $c(\cdot ,t,\tau )$ specifies the instantaneous internal activity decorating the scaffold.

### Geometric symmetry and decorated symmetry

The geometry $\mathcal {S}_{t}$ induces equivalence classes of vertices according to their diffusion-geometric signatures. Let

4.19 $$\begin{aligned} \Pi _{\textrm{geo}}(t)=\left\{ \mathcal {C}_{1}(t),\ldots ,\mathcal {C}_{r}(t)\right\} \end{aligned}$$

denote the geometric partition. Two vertices may be geometrically equivalent while carrying different internal states. Consequently, the decorated object can have lower symmetry than the scaffold.

For every geometric class $\mathcal {C}_{\ell }(t)$, define its mean decoration by

4.20 $$\begin{aligned} \overline{c}_{\ell }(t,\tau )=\frac{1}{|\mathcal {C}_{\ell }(t)|}\sum _{v\in \mathcal {C}_{\ell }(t)}c_{v}(t,\tau ). \end{aligned}$$

We then define the decorated-symmetry error

4.21 $$\begin{aligned} \mathcal {E}_{\textrm{sym}}(t,\tau )=\frac{1}{n}\sum _{\ell =1}^{r}\sum _{v\in \mathcal {C}_{\ell }(t)}\left[ c_{v}(t,\tau )-\overline{c}_{\ell }(t,\tau )\right] ^{2}. \end{aligned}$$

The index vanishes precisely when all vertices belonging to the same geometric class carry identical decorations:

4.22 $$\begin{aligned} \mathcal {E}_{\textrm{sym}}(t,\tau )=0. \end{aligned}$$

For the global temporal analysis, we also use the mean decorated-symmetry error

4.23 $$\begin{aligned} \overline{\mathcal {E}}_{\textrm{sym}}(t)=\frac{1}{N_{\tau }}\sum _{k=1}^{N_{\tau }}\mathcal {E}_{\textrm{sym}}(t,\tau _{k}), \end{aligned}$$

where $N_{\tau }$ is the number of sampled internal times.

For the two-triangle graph, the principal automorphic classes are formed by the four peripheral vertices and the two bridge vertices. Before synchronization, geometrically equivalent vertices may display different activities and $\mathcal {E}_{\textrm{sym}}(t,\tau )$ is generally positive. As propagation suppresses disagreement, the activity values within every class converge and the decorated-symmetry error approaches zero.

At exact equality, the distinction can be expressed through a decorated equivalence relation. Vertices satisfy $i\sim _{\textrm{dec}}j$ if and only if

4.24 $$\begin{aligned} \textbf{s}_{i}^{\textrm{geo}}(t)=\textbf{s}_{j}^{\textrm{geo}}(t) \end{aligned}$$

and

4.25 $$\begin{aligned} c_{i}(t,\tau )=c_{j}(t,\tau ). \end{aligned}$$

This relation defines a partition that refines the exact geometric partition. Pairwise tolerance tests provide numerical proximity criteria; constructing an equivalence relation additionally requires transitivity. At finite numerical resolution, a partition must be obtained with an explicit clustering convention applied within the geometric classes. The reported continuous indices $\mathcal {E}_{\textrm{dis}}$ and $\mathcal {E}_{\textrm{sym}}$ are calculated directly from the activities and geometric classes. As synchronization makes all activities within each geometric class equal in the limit, the limiting decorated partition agrees with the geometric partition.

### From heterogeneous to homogeneous decoration

For small propagation times, the disagreement component remains large and the vertices carry heterogeneous activities. The scaffold already encodes the relational structure of the graph, but its decoration distinguishes some geometrically equivalent vertices. In this regime,

4.26 $$\begin{aligned} \mathcal {E}_{\textrm{dis}}(t,\tau )>0,\qquad \mathcal {E}_{\textrm{sym}}(t,\tau )>0 \end{aligned}$$

for generic internal activity patterns.

As *t* increases, the disagreement component decays:

4.27 $$\begin{aligned} \textbf{v}(t,\tau )=e^{-t\mathscr {L}}\textbf{v}(0,\tau )\longrightarrow \textbf{0}. \end{aligned}$$

Therefore,

4.28 $$\begin{aligned} c_{v}(t,\tau )\longrightarrow \overline{c}(t,\tau )\qquad \text {for every }v\in V. \end{aligned}$$

The decoration becomes spatially homogeneous for each fixed internal time *τ*, and

4.29 $$\begin{aligned} \mathcal {E}_{\textrm{dis}}(t,\tau )\longrightarrow 0,\qquad \mathcal {E}_{\textrm{sym}}(t,\tau )\longrightarrow 0. \end{aligned}$$

**Fig. 5:**
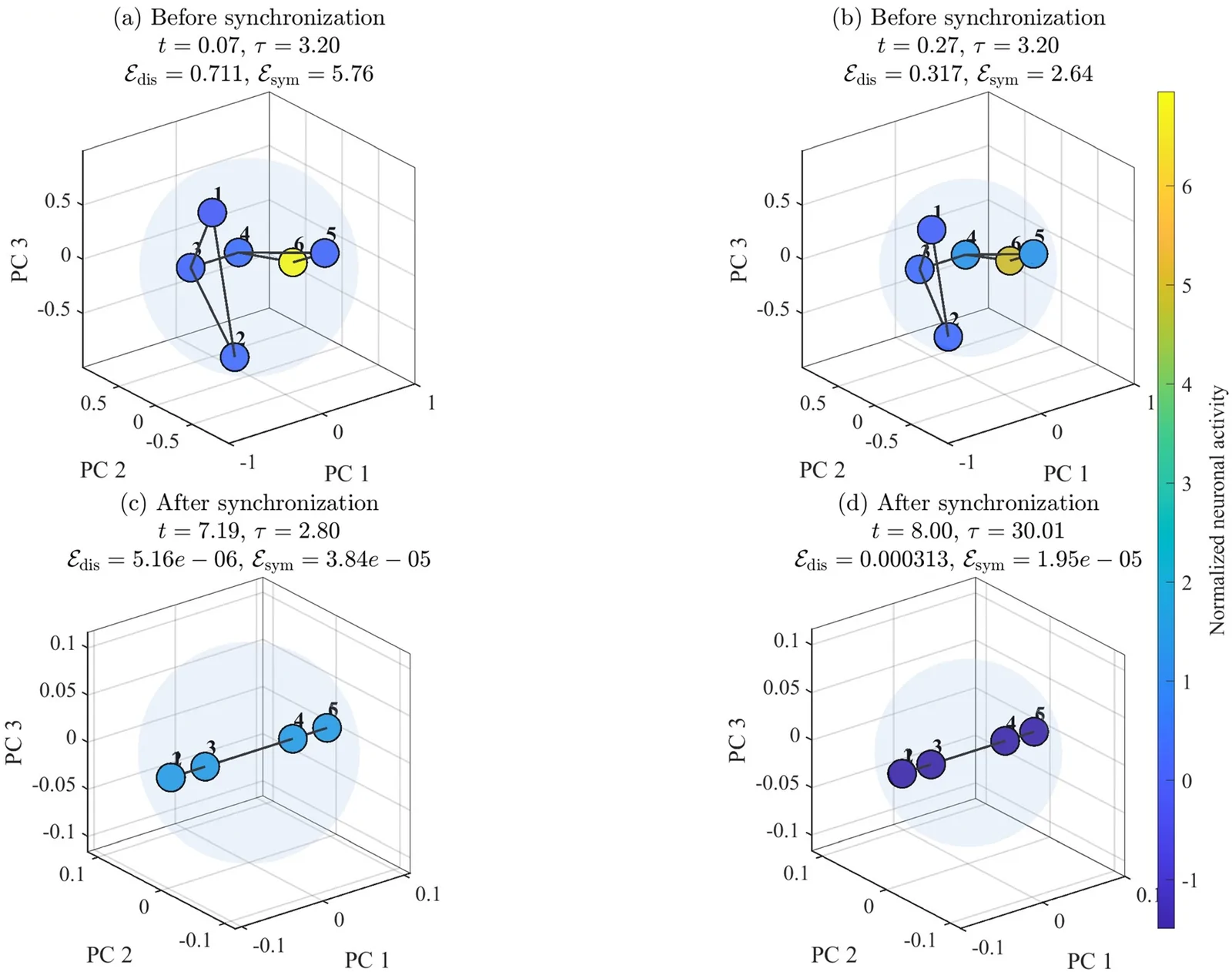
Dynamically decorated diffusion scaffolds before and after synchronization. The three displayed coordinates use the same fixed Laplacian eigenbasis in every panel, and common axis limits are used within each row so that geometric contraction remains visible across panels. Panels (**a**) and (**b**) use the same internal time, *τ =3.20*, at propagation times *t=0.07* and *t=0.27*, respectively. Their different vertex colors therefore show how propagation reorganizes one fixed heterogeneous activity pattern before synchronization. Panels (c) and (d) show the synchronized regime at *(t,τ )=(7.19,2.80)* and (8.00, 30.01), respectively. The decoration is nearly homogeneous within each late panel, but its common value differs between the panels because the internal neuronal waveform continues to evolve with *τ*. The late-time geometries differ because they are evaluated at different propagation times; the common coordinate system reveals the additional contraction from *t=7.19* to *t=8.00*. Panel titles report the corresponding values of $\mathcal {E}_{\textrm{dis}}$ and $\mathcal {E}_{\textrm{sym}}$. The parula colors encode normalized neuronal activity. The translucent spheres provide visual envelopes of the scaffolds

The scaffold itself still changes with diffusion scale *t*, because $\mathcal {S}_{t}$ is generated by $e^{-t\mathscr {L}}$. The invariance under the addition of the consensus nonlinearity concerns the equality between the semilinear and linear scaffolds evaluated at the same *t*. Synchronization acts on the decoration, whereas changing *t* also changes the scale at which the relational geometry is read. Figure 5 illustrates these two simultaneous effects: the progressive homogenization of the neuronal decoration and the contraction of the diffusion scaffold as the propagation scale *t* increases.

### Interpretation within GDT

The decorated construction separates three levels that would otherwise be easily conflated. The instantaneous neuronal state determines what activity is locally present. Diffusion-like propagation organizes the vertices into an integrated relational scaffold. A perceptual coordinate, when present, specifies the particular content instantiated within the corresponding modality.

The complete state proposed by GDT combines geometry, content, and neuronal activity. Two systems may share the same diffusion-induced scaffold while differing in their internal activity decorations or in the perceptual coordinate attached to that scaffold. Conversely, similar instantaneous activity patterns may be embedded in different propagation geometries and therefore participate in different relational organizations.

This distinction refines the relation between unity and differentiation. The scaffold provides geometric unity because all vertices belong to a common metric organization. The decoration provides state-dependent differentiation because geometrically related vertices may carry different activities. Propagation progressively integrates the decoration, while the geometric scaffold determines the relational structure within which that integration occurs.

The present calculation tests the mathematical and computational components of this proposal. It verifies that the consensus-directed nonlinearity leaves the disagreement propagator, and hence the diffusion geometry at each fixed propagation time, unchanged. The computed aggregate disagreement and symmetry errors both decay toward zero, while the semilinear and linear diffusion distances agree to within numerical precision (the maximum discrepancy is below $10^{-10}$). Within GDT, conscious experience additionally requires that the differentiated geometry be intrinsically accessible and recursively used by the system.

The resulting hierarchy may be summarized as

4.30 $$\begin{aligned} \begin{aligned}\text {internal activity}&\longrightarrow \text {network propagation}\longrightarrow \text {diffusion scaffold}\\&\longrightarrow \text {dynamical decoration}\longrightarrow \text {integrated decorated state}. \end{aligned} \end{aligned}$$

Section 3 used this framework to distinguish colorness from a particular color content. The present section extends it by showing how temporally evolving neuronal activity decorates a common diffusion-geometric construction. The simulations instantiate the internal dynamics, evaluate $\mathcal {E}_{\textrm{dis}}(t,\tau )$ and $\mathcal {E}_{\textrm{sym}}(t,\tau )$, and visualize the transition from heterogeneous to nearly homogeneous decoration. Experimental tests of the proposed relation between decorated geometry and conscious content remain a separate task.

## color-content geometry on a human structural connectome

We now combine the scaffold and neuronal-decoration constructions on a human structural connectome. The numerical question is whether a learned distributed encoding, followed by anatomical propagation, can reproduce color relations for stimuli excluded from fitting. CIEDE2000 supplies a controlled perceptual reference. We first estimate the encoding on 16 colors, then freeze it and evaluate 256 new colors. A separate neuronal calculation tests how the learned geometry is expressed after nonlinear stimulus encoding.

### Construction of the human region-level network

The anatomical substrate is the weighted network obtained from the Budapest Reference Connectome and braingraph.org resources (Szalkai et al. 2017; Kerepesi et al. 2017). The supplied high-resolution graph contains 480 anatomically labelled vertices, assigned by FreeSurfer labels to 76 macroscopic regions. Connections between distinct regions are aggregated as

5.1 $$\begin{aligned} W_{rs}=\sum _{\begin{array}{c} i:\pi (i)=r\\ j:\pi (j)=s \end{array} }A_{ij}^{(480)},\qquad r\ne s, \end{aligned}$$

with $W_{rr}=0$ and numerical symmetrization $\mathscr {W}\leftarrow (\mathscr {W}+\mathscr {W}^{T})/2$. The resulting graph is connected and contains 265 undirected edges. Dividing positive weights by their mean gives $\widetilde{\mathscr {W}}$. We use

5.2 $$\begin{aligned} \mathscr {L}_{0}=\operatorname {diag}(\widetilde{\mathscr {W}}\textbf{1})-\widetilde{\mathscr {W}},\qquad \mathscr {L}=\frac{\mathscr {L}_{0}}{\lambda _{2}(\mathscr {L}_{0})}. \end{aligned}$$

Thus *t* is dimensionless and measured relative to the slowest nonconstant relaxation mode.

Diffusion-MRI tractography estimates macroscopic pathways indirectly. Crossing fibers and other complex configurations can produce ambiguous reconstructions, false-positive connections, and missed pathways (Maier-Hein et al. 2017). The undirected regional weights summarize structural connectivity, and the Laplacian supplies an effective propagation model at that scale. Axonal direction, synaptic physiology, and calibration to physiological latency are further levels of biological specification.

### Cortical support and learned encoding

The encoding uses the bilateral pericalcarine, lateral occipital, lingual, and fusiform cortices, spanning early visual, extrastriate, and ventral occipitotemporal regions implicated in chromatic processing (Gegenfurtner 2003; Conway et al. 2007; Brouwer and Heeger 2009; Lafer-Sousa and Conway 2013). Let $\mathscr {B}_{0}\in \mathbb {R}^{76\times 3}$ be an orthonormal basis for vectors supported on these eight regions, equal within each bilateral pair, and centered over the support. This subspace has dimension three. The learned encoding is

5.3 $$\begin{aligned} \mathscr {B}=\alpha \mathscr {B}_{0}F,\qquad F\in \mathbb {R}^{3\times 3},\qquad \operatorname {Tr}(\mathscr {B}^{T}\mathscr {B})=3, \end{aligned}$$

where *F* is full rank and *α* fixes the total encoding norm. Its gains and channel mixing are estimated from training colors. Centering, bilateral equality, and regional support are preserved, while the learned columns can have different norms and inner products.

This replaces the initial heuristic regional coefficients with a reproducible estimation rule. The anatomical choice specifies where the model represents color; the numerical weights specify how the three color coordinates are distributed within that support. Supplementary Information (SI), Sections S1–S2, give the fitted coefficients, objective function, and validation protocol.

### Training and new-color ensembles

The 16 training colors comprise lightness values $L^{*}\in \{45,65\}$, hue angles $h_{ab}\in \{25^{\circ },115^{\circ },205^{\circ },295^{\circ }\}$, and chroma fractions 0.45 and 0.75 of the largest sRGB-valid chroma at each lightness and hue under D65. Each stimulus has CIELAB coordinate $\textbf{p}_{s}$ and centered coordinate $\boldsymbol{\xi }_{s}=\textbf{p}_{s}-(60,0,0)^{T}$.

We first use 40 balanced eight-training/eight-test splits within this ensemble. The fitting and regularization choices use training colors exclusively. The final encoding is then estimated from all 16 colors and frozen. A new ensemble of 256 colors samples lightness uniformly on [45, 65], hue uniformly on [0, 360), and the gamut-relative chroma fraction uniformly on [0.45, 0.75]. These new colors provide 32,640 pairwise comparisons. Their generation and subsequent evaluation use the frozen encoding and propagation time. SI Sections S2–S3 specify the sampling, including the random seeds.

### Induced metric and estimation

With $\mathscr {K}(t)=e^{-t\mathscr {L}}$, the encoded response and its content metric are

5.4 $$\begin{aligned} \textbf{x}_{s}(t)=\mathscr {K}(t)\mathscr {B}\boldsymbol{\xi }_{s},\qquad \mathscr {G}_{\mathcal {P}}(t)=\mathscr {B}^{T}e^{-2t\mathscr {L}}\mathscr {B}. \end{aligned}$$

For $$\boldsymbol{\delta }_{sr}=\textbf{p}_{s}-\textbf{p}_{r}$$,

5.5 $$\begin{aligned} D_{sr}^{\textrm{neur}}(t)=\sqrt{\boldsymbol{\delta }_{sr}^{T}\mathscr {G}_{\mathcal {P}}(t)\boldsymbol{\delta }_{sr}}. \end{aligned}$$

The factor 2*t* follows from comparing responses propagated for duration *t*: their Gram operator is $\mathscr {K}(t)^{T}\mathscr {K}(t)$. At every finite time the metric is positive definite, since the heat kernel is invertible and the encoding has full column rank.

The representative duration remains $t_{0}=0.023$, fixed before estimation of the revised encoding. It originally selected approximately half of the metric trace for the initial orthonormal encoding. With the learned encoding, the same duration retains *39.75%* of its initial trace. We therefore treat $t_{0}$ as a fixed computational propagation scale. SI Section S5 reports the results at additional durations.

To estimate the encoding, we fit a positive-definite color metric *H* by regularized squared-distance regression against CIEDE2000. Writing

5.6 $$\begin{aligned} G_{0}=\mathscr {B}_{0}^{T}e^{-2t_{0}\mathscr {L}}\mathscr {B}_{0},\qquad F=G_{0}^{-1/2}H^{1/2}, \end{aligned}$$

gives $\mathscr {G}_{\mathcal {P}}(t_{0})=\alpha ^{2}H$. The common factor $\alpha ^{2}$ preserves all distance ranks. The estimation objective balances agreement with the training reference against an isotropic penalty $\lambda \Vert H-I\Vert _{F}^{2}$. Nested validation selects *λ* using training colors only. The final selected value is 0.001.

The continuous metric is spatially constant and hence flat; its eigenvalues describe directional scaling of color differences. The finite vertex scaffold retains its separate circum-Euclidean interpretation. Together, the encoding and propagator connect these two relational domains.

### Prediction of color relations

Table 1 summarizes agreement with CIEDE2000. The first column averages correlations over the eight held-out colors in each balanced split. The second uses the 256 new colors after the final encoding has been frozen. Direct CIELAB distances provide the colorimetric baseline. The initial heuristic encoding is retained as a methodological comparison.

**Table 1:** Spearman correspondence with CIEDE2000

Representation	Held-out mean	New colors
Initial encoding, propagated	0.6231	0.7908
Learned encoding, propagated	0.8300	0.9190
Learned encoding, before propagation	0.7631	0.8575
Direct CIELAB	0.8204	0.9229

The repeated-split scores use 28 test pairs per split; the new-color scores use 32,640 pairs

Learning substantially improves the propagated representation relative to the initial weights. The improvement transfers to the new-color ensemble, where the model closely approaches the colorimetric baseline. In the repeated-split analysis, the learned propagated representation exceeds direct CIELAB on 29 of 40 splits. These splits share stimuli and describe exploratory within-ensemble predictive performance; the new-color test supplies the separate frozen-model evaluation.

For the new colors, conditional stimulus-bootstrap *95%* intervals are [0.9014, 0.9361] for the learned propagated representation and [0.9072, 0.9385] for direct CIELAB. Their paired difference is *-0.0039*, with interval *[-0.0154,0.0075]*. These intervals quantify resampling variability within the new-color ensemble under the fixed model. The purpose of the comparison is to assess retention of perceptual organization through encoding and propagation. SI Section S6 gives the resampling method.

### Nonlinear neuronal realization

We reran the Izhikevich encoding with the learned regional weights, retaining the original neuronal parameters (Izhikevich 2003, 2007). Each region has a local regular-spiking unit:

5.7 $$\begin{aligned} \frac{dv_{r}}{d\tau }=0.04v_{r}^{2}+5v_{r}+140-u_{r}+I_{r}(\tau ),\qquad \frac{du_{r}}{d\tau }=a_{r}(b_{r}v_{r}-u_{r}), \end{aligned}$$

with the standard reset $v_{r}\leftarrow c_{r}$, $u_{r}\leftarrow u_{r}+d_{r}$ at $v_{r}\ge 30$. Small deterministic regional variations specify $a_{r},b_{r},c_{r},d_{r}$ and baseline current. For stimulus *s*,

5.8 $$\begin{aligned} I_{r,s}(\tau )=I_{r}^{(0)}+9\,g(\tau )\frac{(\mathscr {B}\boldsymbol{\xi }_{s})_{r}}{z_{\max }}, \end{aligned}$$

where *g=1* from 100 to 500 ms and zero otherwise. The normalization $z_{\max }=41.1541$ is determined from the 16 training colors and frozen for the new-color calculation. Simulation lasts 600 ms, with step 0.5 ms and an 18-ms exponential rate filter. All regional parameters and averaging windows are specified in SI Section S4.

The baseline-corrected mean evoked rates form $\textbf{r}_{s}$. Their propagated, centered representations are

5.9 $$\begin{aligned} \textbf{y}_{s}=\mathscr {P}e^{-t_{0}\mathscr {L}}\textbf{r}_{s},\qquad \mathscr {P}=I-\frac{1}{76}\textbf{1}\textbf{1}^{T}. \end{aligned}$$

Thus local nonlinear encoding is followed by the same structural propagation used in the analytic construction. Pairwise distances between $\textbf{y}_{s}$ define $D^{\textrm{dyn}}$.

For the 256 new colors, the analytic–neuronal correspondence is 0.9244, with conditional *95%* interval [0.9098, 0.9382]. The neuronal correspondence with CIEDE2000 is 0.8199, with interval [0.7945, 0.8465]. For the 16 fitting colors, the respective values are 0.8821 and 0.7830. These newly computed results express the learned geometry through thresholding, resets, heterogeneous neuronal parameters, and temporal filtering. The analytic–neuronal comparison assesses agreement between two model representations, while the CIEDE2000 comparison assesses their relation to the perceptual reference.

### Interpretation within GDT

The learned encoding gives the model a constructive relation to color content: anatomical propagation generates a metric whose ordering closely follows the perceptual reference for new stimuli. The neuronal calculation shows that much of this relational organization remains expressed after nonlinear encoding. This supplies a candidate mechanism for the relational organization of color experience within GDT.

Learning is an explicit modeling assumption. The factorization through *H* separates the fitted content metric from its anatomical realization: positive-definite metrics can also be realized through other full-rank encodings and propagators. Consequently, these calculations establish the representational capability of the encoding–propagation combination. Anatomical selection, intrinsic access, and recursive causal use remain separate components of the GDT hypothesis. SI Section S7 records the initial-encoding controls and explains their relation to the revised model.

## Discussion: from signals and decorations to intrinsically accessible geometry

The three constructions connect diffusion geometry to color content at complementary levels. The elementary graph separates the modality-level scaffold from particular color coordinates. The semilinear example establishes how neuronal decoration can change on a preserved scaffold. The human-connectome model adds learned regional encoding and evaluation on new colors. Together, they specify a candidate relational organization of color content within GDT.

Central to this account is the distinction between two related geometries. The scaffold $\mathcal {S}_{t}$ describes relations among substrate elements. A distributed encoding $\Phi (\textbf{p})=\mathscr {B}(\textbf{p}-\textbf{p}_{0})$ induces a second geometry on the content domain:

6.1 $$\begin{aligned} \mathscr {G}_{\mathcal {P}}(t)=\mathscr {B}^{T}e^{-2t\mathscr {L}}\mathscr {B}. \end{aligned}$$

The two objects share the propagation operator but have different domains: vertices for the scaffold, possible contents for the induced metric. Their composition makes explicit how the same anatomical substrate can support multiple particular colors within a common relational organization.

Within this organization, a realized response $\textbf{c}(t)=\mathscr {K}(t)\textbf{c}_{0}$ describes the state generated by one input. The full response family determines the relations among possible states. In the elementary example, arithmetic consensus decodes a particular color; in the connectome example, a specified color is encoded across eight cortical regions. More generally, a distributed decoding relation can be written

6.2 $$\begin{aligned} \textbf{p}(\tau )=\Psi _{\mathcal {P}}(\text {sensory input},\text {internal neuronal state}). \end{aligned}$$

Here $\Psi _{\mathcal {P}}$ denotes a physical correspondence between activity and content. Different neuronal states can represent the same content, while systematic changes in content require corresponding physical differences.

The scaffold, its induced content metric, the particular content, and its neuronal decoration can therefore be combined in the structured object

6.3 $$\begin{aligned} \mathfrak {Q}_{t,\tau }=\left( \mathcal {S}_{t},\mathscr {G}_{\mathcal {P}}(t),\textbf{p}(\tau ),c(\cdot ,t,\tau )\right) . \end{aligned}$$

The coordinate specifies the represented color, the metric specifies its relations to other possible colors, and the decoration describes the contemporaneous neural activity. Within GDT, this structured object acquires its proposed experiential interpretation through intrinsic access and recursive causal use. This connects the observational representation of a particular color with the theory’s account of its felt qualitative character.

The human-connectome calculation gives this construction a quantitative realization by learning a distributed encoding and testing it on new colors. Its 0.9190 correspondence with CIEDE2000 approaches the direct CIELAB value 0.9229, while its analytic–neuronal correspondence is 0.9244. These are complementary results: the first concerns representation of the perceptual reference; the second concerns expression of the model geometry through nonlinear neuronal encoding. Spearman correlation measures ordering, so similar ranks can coexist with nonuniform changes in distance magnitudes.

The achievement is therefore a reproducible mechanism that transfers learned color relations to new stimuli through an anatomical propagation model. The factorization of the learned metric identifies the anatomical realization as one component of this mechanism. Independent biological constraints on encoding and access would further specify its interpretation within GDT.

These results also illustrate how relational unity and differentiation coexist: the common metric configuration organizes the content domain, while positions within it distinguish particular contents. Neuronal heterogeneity supplies a further form of differentiation. Spatial synchronization can homogenize the decoration while leaving its common waveform temporally variable; changes in propagation duration can simultaneously contract the scaffold and alter the induced content metric. These distinctions explain how integrated geometry and varied activity can coexist.

The proposed transition from this relational organization to intrinsically used geometry calls for mechanisms through which its relations influence further processing. Recurrent propagation, convergent pathways, distributed comparison, synaptic integration, and activity-dependent feedback provide candidates for this role. We denote this functional capacity by

6.4 $$\begin{aligned} \mathfrak {R}:\{\text {propagation responses}\}\longrightarrow \{\text {causally effective relational variables}\}. \end{aligned}$$

The neuronal calculation specifies relational information available in the model’s activity. GDT proposes that intrinsic access and recursive use connect such organization to qualitative experience. This proposal concerns the system’s own use of the relations; the mathematical construction supplies their explicit form.

The explanatory question of why a causally effective organization has subjective character remains distinct from its functional specification. Predictive coding and cortical gradients help develop the computational and neurobiological mechanisms through which the proposed relational organization could be used.

In particular, predictive coding suggests how a response geometry could contribute to anticipation and comparison. In the visual model of Rao and Ballard (1999), descending signals convey expected activity and ascending signals convey residual errors. Friston and Kiebel (2009) extend this principle to hierarchical dynamical generative models: a current inferred state is distinguished from the family of trajectories organized by the model. This distinction is structurally related to the observational and geometric levels of GDT. A particular response instantiates one state or trajectory; the operator and its induced geometry characterize relations across the available response repertoire. This correspondence relates a realized representation to the organization constraining possible representations.

For the fixed propagation law considered here, the semigroup property gives

6.5 $$\begin{aligned} \textbf{c}(t+\Delta )=\mathscr {K}(\Delta )\textbf{c}(t),\qquad \mathscr {K}(\Delta )=e^{-\Delta \mathscr {L}}. \end{aligned}$$

The kernel at a specified horizon determines responses to all initial states at that horizon; the family $\{\mathscr {K}(\Delta ):\Delta \ge 0\}$ specifies their continued evolution. Under the model, proximity of response profiles means similarity of their predicted distributed consequences. If a system uses these relations to anticipate and compare responses, its diffusion geometry could supply part of the structure of an internal predictive model. This predictive interpretation assigns a causal role to the internal use of response relations.

A precise mathematical connection is provided by predictive maps. Stachenfeld et al. (2017) represent a state through discounted expected future occupancies. For a transition matrix $\mathscr {T}$ and *0<γ <1*, the successor operator is

6.6 $$\begin{aligned} \mathscr {M}_{\gamma }=\sum _{k=0}^{\infty }\gamma ^{k}\mathscr {T}^{k}=(\mathscr {I}-\gamma \mathscr {T})^{-1}. \end{aligned}$$

For continuous-time diffusion on the present undirected nonnegative network, $-\mathscr {L}$ is a Markov generator. Its discounted occupancy analogue is

6.7 $$\begin{aligned} \mathscr {R}_{\alpha }=\int _{0}^{\infty }e^{-\alpha s}e^{-s\mathscr {L}}\,ds=(\alpha \mathscr {I}+\mathscr {L})^{-1},\qquad \alpha>0. \end{aligned}$$

The identity follows by integrating each Laplacian eigenmode. The heat kernel and this resolvent share eigenvectors, weighted by $e^{-s\lambda _{k}}$ and $(\alpha +\lambda _{k})^{-1}$, respectively. Thus, fixed-horizon response geometry and a temporally integrated predictive representation arise from the same generator through different spectral weightings. Environmental state transitions in the predictive-map model and anatomical propagation in GDT are different substrates; the connection is the common organization of states through their possible future consequences.

These relations suggest a concrete extension of intrinsic access. Recurrent circuits could compare expected and input-evoked response profiles, while learning modifies effective propagation or encoding. The present model specifies a geometry on which such comparisons could act. Learning, precision weighting, and prediction-error updates provide concrete mechanisms for extending it into a predictive architecture. A test would ask whether propagation geometry estimated independently of a task predicts similarity among anticipatory responses or the pattern of violations of learned expectations, beyond current activation and stimulus similarity.

The organization of such predictive representations can also be considered at the scale of cortical gradients, which supplies a complementary anatomical and empirical context. Margulies et al. (2016) used diffusion embedding to identify a principal connectivity axis from sensorimotor to transmodal cortex. Zhang et al. (2019) described interdependent gradients and related their organization to the maintenance and updating of an internal model. Using diffusion-map embeddings, Katsumi et al. (2023) found corresponding association–sensorimotor and representation–modulation gradients across isocortex, cerebellum, and hippocampus. They interpret these axes in terms of predictive representations and their regulation, while distinguishing measured connectivity correspondence from a hypothesis about directed signal flow.

The shared mathematical principle is to describe a region by its relations across a network. In GDT, if $\mathscr {L}\textbf{u}_{k}=\lambda _{k}\textbf{u}_{k}$, response distances at propagation duration *s* satisfy

6.8 $$\begin{aligned} \left\| e^{-s\mathscr {L}}(\textbf{e}_{i}-\textbf{e}_{j})\right\| ^{2}=\sum _{k}e^{-2s\lambda _{k}}\bigl (u_{k}(i)-u_{k}(j)\bigr )^{2}. \end{aligned}$$

This expression links the geometry to structural connectome harmonics, which provide a spatial basis for neural activity (Atasoy et al. 2016). Propagation determines how strongly each mode contributes to the metric; increasing *s* suppresses rapidly decaying modes relative to slower ones. With the scaffold convention of Section 2.3, the left-hand side equals $\mathscr {D}^{2}(2s;i,j)$.

Functional diffusion embeddings generally operate on similarities between connectivity profiles, whereas the present heat kernel operates on a structural graph. The relation between their respective modes is an empirical question. Gradient observations motivate testing whether dominant functional axes align with structural response modes, and whether task-dependent changes in this alignment accompany changes in representational geometry. Gradients also complement a parcellated substrate: discrete regions can occupy continuously varying coordinates along functional axes.

Activity-flow modeling offers a further link. Cole et al. (2016) predicted held-out task activations using connectivity-weighted activity in other regions, supporting a role for network organization in shaping distributed responses. Together with the gradient results, this finding motivates the GDT hypothesis that a common relational scaffold can constrain both internally anticipated and stimulus-evoked activity. The representation–modulation distinction further suggests candidate interactions through which relational information could be selected and causally used. Measuring the relevant activity flows and their causal consequences would test this interpretation.

## Empirical predictions

The constructions developed in this paper lead to three testable predictions. The present work establishes the mathematical construction, implements it on a human structural connectome, evaluates its learned encoding on new colors, and quantifies its relational organization after nonlinear neuronal realization. It thereby provides a concrete empirical programme for testing the GDT hypothesis that the relational organization of qualitative content corresponds to geometry instantiated by neural dynamics and intrinsically accessible to the system that generates it.

1. The first concerns the relation between subjective color experience and neural propagation geometry. As proposed in Section 3.7, a controlled ensemble of color stimuli can be used to construct a subjective dissimilarity matrix $\textbf{D}^{\textrm{subj}}$ from pairwise similarity or dissimilarity judgments. This matrix can then be compared with the distance matrix $\textbf{D}^{\textrm{act}}$ obtained from measured neural activation patterns, the colorimetric reference matrix $\textbf{D}^{\textrm{CIELAB}}$, and the distance matrix $\textbf{D}^{\textrm{diff}}$ generated by an empirically estimated neural propagation geometry. The principal prediction is that $\textbf{D}^{\textrm{diff}}$ explains variability in $\textbf{D}^{\textrm{subj}}$ beyond that explained by $\textbf{D}^{\textrm{act}}$ and $\textbf{D}^{\textrm{CIELAB}}$. This can be tested using representational-similarity analysis, partial matrix correlations, regression models, and cross-validated model comparisons. The prediction distinguishes GDT from the more general observation that neural responses and perceptual judgments can both be represented geometrically: it assigns a specific explanatory role to the relations generated by propagation.
2. The second prediction concerns propagation scale. The human-connectome calculation shows that the model–reference correspondence $\rho _{\textrm{per}}(t)$ changes systematically with *t* as the principal directions of the induced content metric contract at different rates. Neural–perceptual correspondence should therefore depend on the temporal or spatial scale over which neural communication is integrated. GDT motivates testing an intermediate regime in which distributed relations are available before differentiation is strongly attenuated. This hypothesis concerns subjective and neural data. SI Section S5 reports how the revised model depends on propagation duration with the learned encoding held fixed. Empirically estimating this dependence would test whether subjective color relations are associated with a particular range of neural propagation scales.
3. The third prediction concerns the distinction between changes in content and changes in geometry. Different colors can occupy different positions within a common content geometry while sharing the same substrate and propagation operator. Conversely, changes in effective connectivity or propagation dynamics can deform the content geometry even when the external stimulus coordinates remain unchanged. The framework therefore predicts that experimentally manipulating the stimulus and manipulating neural propagation should have distinguishable effects: the former should primarily change the instantiated position within the geometry, whereas the latter should reorganize the relations among possible contents.

## Conclusions

The paper develops a candidate account of color relations within GDT by connecting a diffusion-induced scaffold, distributed content encoding, and neuronal realization. The learned encoding transfers to new colors: after anatomical propagation its correspondence with CIEDE2000 is 0.9190, close to the direct CIELAB reference of 0.9229. The rerun neuronal model gives analytic–neuronal correspondence of 0.9244. These results establish a concrete way in which anatomical propagation can support a perceptually organized geometry whose relations remain strongly expressed under nonlinear encoding.

The central GDT proposal concerns the role of this organization when its relations are intrinsically accessed and recursively used. Predictive coding, cortical gradients, and activity-flow models supply possible mechanisms for such use and guide empirical comparisons. The construction and new-color evaluation advance the proposed relational account of color content; its connection to experienced color similarities and to subjective character remains the subject of the theory’s empirical and explanatory questions.

## Supplementary Information

Below is the link to the electronic supplementary material.

Supplementary file 1 (pdf 249 KB)

## Data Availability

The human-connectome data used in this study are publicly available through the Budapest Reference Connectome and braingraph.org resources cited in the manuscript. The numerical results and MATLAB code supporting the findings are available from the corresponding author on reasonable request.
