# Supplementary material for: Can qualia be geometric? A geometro-diffusion framework for the relational structure of color experience
Source: Cogn Neurodyn. 2026 Nov 7;20(1):216. doi: 10.1007/s11571-026-10577-2 (PMC13744580; doi:10.1007/s11571-026-10577-2)
Supplement: Supplementary file 1 [file 11571_2026_10577_MOESM1_ESM.pdf]

SUPPLEMENTARY INFORMATION. CAN QUALIA BE GEOMETRIC?

ERNESTO ESTRADA

S1. Encoding and estimation

The structural substrate is the 76-region, 265-edge undirected weighted connectome described in Section 5.1 of the main manuscript. We retain the bilateral pericalcarine, lateral occipital, lingual, and fusiform support. An auxiliary orthonormal basis B_0 spans the bilaterally equal, support-centered vectors. For reproducibility, its seed rows are $(1, 0, 0)$, $(0.50, 0.20, 0.65)$, $(0.20, 0.55, 1)$, and $(0, 1, 0.20)$ for these four regions, respectively, duplicated across hemispheres. Subtracting each column's mean on the eight-region support and applying modified Gram–Schmidt gives B_0. It is zero elsewhere.

At the fixed duration $t_0 = 0.023$, let $G_0 = B_0^T e^{-2t_0 L} B_0$. For a positive-definite learned metric H, define

$$F = G_0^{-1/2} H^{1/2}, \qquad B = \alpha B_0 F, \qquad \alpha = \sqrt{\frac{3}{\mathrm{Tr}(F^T F)}}. \tag{S1.1}$$

Then $B^T e^{-2t_0 L} B = \alpha^2 H$ and $\mathrm{Tr}(B^T B) = 3$. Table S1 gives the resulting coefficients. Each row applies to both hemispheres; all other regional rows are zero. The slight rounding in the table is solely for display.

Table S1. Learned regional encoding coefficients; each row is used bilaterally.

Region	L^* channel	a^* channel	b^* channel
Pericalcarine	0.6133265462	0.3309716619	0.1056527427
Lateral occipital	0.0703996294	−0.5991767692	−0.3255269615
Lingual	−0.2201000089	−0.1550874076	0.2555304883
Fusiform	−0.4636261667	0.4232925149	−0.0356562695

For a training set with m distinct color pairs, write $\delta_{sr} = p_s - p_r$, and let s_x and s_y be the means of $\|\delta_{sr}\|^2$ and $(D_{sr}^{00})^2$ within that set. We estimate

$$\widehat{H}_\lambda = \underset{H \succeq 10^{-6} I}{\mathrm{argmin}} \left\{ \frac{1}{m} \sum_{s<r} \left(\frac{\delta_{sr}^T H \delta_{sr}}{s_x} - \frac{(D_{sr}^{00})^2}{s_y} \right)^2 + \lambda \|H - I\|_F^2 \right\}. \tag{S1.2}$$

The objective is a convex quadratic in the six independent symmetric-matrix entries. We use the coordinates $(H_{11}, H_{22}, H_{33}, \sqrt{2}H_{12}, \sqrt{2}H_{13}, \sqrt{2}H_{23})$, so their Euclidean norm equals

the matrix Frobenius norm. The algorithm starts from the unconstrained ridge solution, clips eigenvalues below 10^{-6}, and uses projected-gradient steps with step size equal to the inverse largest eigenvalue of the quadratic coefficient matrix. Iteration stops at relative parameter change below 10^{-10} or 5,000 iterations. The regularization candidates are

$$\lambda \in \{0, 10^{-4}, 10^{-3}, 10^{-2}, 10^{-1}, 1, 10, 100, \infty\}, \tag{S1.3}$$

where $\lambda = \infty$ specifies $H = I$. Common distance scaling is immaterial to Spearman correlation. Regularization favours isotropy when the training comparisons provide weak evidence for a directional deformation.

The final encoding uses $\lambda = 0.001$, selected by internal validation on the 16 fitting colors. Its total squared norm is 3. At t_0 its trace-retention fraction is 0.397525, and its full-fitting-set CIEDE2000 correlation is 0.911070. The latter is a training score. The main manuscript emphasizes the held-out and new-color evaluations.

S2. Nested validation and the frozen model

The original 16 colors are ordered by lightness $45, 65$, then hue $25°, 115°, 205°, 295°$, then relative chroma $0.45, 0.75$. Forty outer splits each contain eight training and eight test colors. Each half contains two colors from each hue, four from each lightness, and four from each relative-chroma level. Table S2 specifies 20 complementary partitions, evaluated in both directions.

Within each outer training set, we leave out every pair of training colors in turn. The remaining six colors supply 15 fitting pairs. The 13 pairs involving at least one inner withheld color supply the inner validation score. For each λ, we average these Spearman scores over all 28 inner splits. Numerical ties favor the larger λ. After selecting λ, the metric is refitted on all eight outer training colors and evaluated on the 28 pairs wholly within the eight outer test colors. Thus the outer test colors enter neither parameter fitting nor regularization selection.

The mean outer-test correlation is 0.830049, compared with 0.623112 for the initial encoding followed by propagation, 0.763109 for the learned encoding before propagation, and 0.820443 for direct CIELAB. The learned propagated representation exceeds direct CIELAB on 29 of the 40 splits. Mean training correlation is 0.894978. The splits share stimuli, and the original ensemble had been examined during model development; these averages characterize exploratory within-ensemble generalization.

For the final model, the same leave-two-colors-out rule selects regularization using all 16 fitting colors: each inner fit uses 14 colors and its validation score uses the 29 pairs involving either withheld color. We then fit all 120 pairs once and freeze B, t_0, and the stimulus-current normalization. The subsequent 256-color ensemble is used exclusively for evaluation.

TABLE S2. One half of each complementary partition. The other half is its complement in $\{1, \ldots, 16\}$. Both training/test directions are evaluated.

Partition	color indices in one half
1	$1, 4, 7, 8, 9, 12, 13, 14$
2	$1, 2, 3, 8, 12, 13, 14, 15$
3	$1, 5, 6, 8, 9, 11, 12, 16$
4	$1, 3, 4, 5, 10, 14, 15, 16$
5	$1, 4, 5, 7, 10, 12, 13, 16$
6	$1, 2, 4, 7, 12, 13, 14, 15$
7	$1, 4, 5, 7, 10, 11, 14, 16$
8	$1, 4, 5, 8, 10, 11, 14, 15$
9	$1, 2, 4, 7, 11, 13, 14, 16$
10	$1, 4, 6, 7, 9, 12, 14, 15$
11	$1, 2, 3, 7, 12, 13, 14, 16$
12	$1, 4, 6, 8, 9, 11, 14, 15$
13	$1, 5, 6, 8, 10, 11, 12, 15$
14	$1, 2, 3, 6, 12, 13, 15, 16$
15	$1, 4, 6, 7, 9, 11, 14, 16$
16	$1, 2, 5, 7, 11, 12, 14, 16$
17	$1, 3, 6, 8, 10, 11, 13, 16$
18	$1, 6, 7, 8, 9, 11, 12, 14$
19	$1, 4, 6, 7, 10, 11, 13, 16$
20	$1, 4, 5, 8, 9, 11, 14, 16$

S3. COLOR GENERATION AND THE PERCEPTUAL REFERENCE

For each lightness L^* and hue h, the maximum D65/sRGB-valid chroma $C_{\max}$ is found by 50 bisection steps on $[0, 180]$. At every step, $(L^*, C \cos h, C \sin h)$ is converted to linear sRGB and accepted when all three channels lie in $[0, 1]$. The original colors use $C = 0.45C_{\max}$ or $0.75C_{\max}$.

The fresh ensemble uses NumPy's PCG64 generator with seed 20261009. In order, it draws 256 lightness values uniformly on $[45, 65]$, 256 hue values on $[0, 360)$, and 256 relative-chroma values on $[0.45, 0.75]$. The resulting coordinates are

$$p_s = (L_s^*, f_s C_{\max}(L_s^*, h_s) \cos h_s, f_s C_{\max}(L_s^*, h_s) \sin h_s). \tag{S3.1}$$

This ensemble samples intermediate lightness and hue values in addition to varying chroma. Direct CIELAB distances and CIEDE2000 differences are evaluated on the same 32,640 pairs. CIEDE2000 uses $k_L = k_C = k_H = 1$ (Sharma et al., 2005); it is a reference dissimilarity. Euclidean distances in the propagated representation supply the model metric.

The fitted model's fresh-color correlation is 0.918961, compared with 0.922866 for direct CIELAB, 0.857517 for its own unpropagated encoding, and 0.790771 for the initial heuristic encoding followed by propagation. No model parameter is changed after this evaluation.

The implementation reproduces the original stored CIEDE2000 values away from exactly opposite hue directions and agrees with standard reference examples. Two pairs in the original factorial ensemble lie on the 180° hue-branch boundary, where floating-point evaluation in MATLAB and NumPy selects different branches. The stored MATLAB target matrix is retained unchanged throughout fitting. The continuously sampled fresh ensemble avoids exact opposite-hue pairs, and its targets use the same stated CIEDE2000 formula.

S4. Neuronal realization with the learned weights

All neuronal parameters are fixed independently of the learned metric. For region index $r = 1, \dots, 76$,

$$\begin{aligned} a_r &= 0.020 + 0.002\sin(2\pi r/76), & b_r &= 0.200 + 0.010\cos(2\pi r/76), \\ c_r &= -65 + 2\sin(4\pi r/76), & d_r &= 8 + 0.6\cos(4\pi r/76), \\ I_r^{(0)} &= 6 + 0.5\sin(6\pi r/76). \end{aligned} \tag{S4.1}$$

Initial conditions are $v_r(0) = -65 + 2\cos(2\pi r/76)$ and $u_r(0) = b_r v_r(0)$. The dynamics and reset are given in Section 5.6 of the main manuscript. We integrate for 600 ms with step $\Delta\tau = 0.5$ ms. Two consecutive half Euler steps update v, followed by one full update of u, spike detection at $v \geq 30$, filtering, and reset. A spike contributes to the exponentially filtered rate as

$$f_r(\tau + \Delta\tau) = e^{-\Delta\tau/18} f_r(\tau) + \left(1 - e^{-\Delta\tau/18}\right) \frac{1000}{\Delta\tau} \, \mathbf{1}_{\{v_r \geq 30\}}. \tag{S4.2}$$

Rates are stored in single precision, matching the original simulation protocol.

The color drive is $B(p_s - (60, 0, 0)^T)$. Its normalization is the largest absolute component across the 16 fitting colors, $z_{\max} = 41.15406944$, and is frozen for the 256 new colors. The latter have maximum normalized magnitude 1.1344; the drive is used directly. The stimulus current has gain 9 and is active from 100 through 500 ms.

For each region, the evoked feature is the mean filtered rate on $150 \leq \tau \leq 500$ ms minus its mean on $50 \leq \tau < 100$ ms. These local features are propagated by $e^{-t_0 L}$ and centered over all regions. The previous simulation was first reproduced with its original encoding, recovering the archived mean rates to numerical precision. The learned-encoding simulations were then rerun for both ensembles.

On the 16 fitting colors, analytic–neuronal correspondence is 0.882068 and neuronal–CIEDE2000 correspondence is 0.782978. On the 256 new colors these are 0.924389 and 0.819936, respectively. Before propagation, the neuronal–CIEDE2000 correlations are 0.864171 and 0.831025. These are comparisons of simulated representations; the analytic–neuronal and model–reference scores answer complementary questions.

S5. Propagation-scale sensitivity

The learned encoding is held fixed while varying the propagation duration. Table S3 uses the 256 new colors and the frozen local neuronal responses. All durations use the same spectrally normalized Laplacian. The original $t_0 = 0.023$ is retained as the primary duration rather than selected from the fresh-color scores.

Table S3. Propagation-scale dependence of the learned representation on new colors. The retention is $\mathrm{Tr}(B^T e^{-2tL} B)/\,\mathrm{Tr}(B^T B)$.

t	Retention	$\rho_{\mathrm{an},00}$	$\rho_{\mathrm{dyn},00}$	$\rho_{\mathrm{an,dyn}}$
0	1.0000	0.8575	0.8310	0.9429
0.008	0.6984	0.8873	0.8458	0.9366
0.023	0.3975	0.9190	0.8199	0.9244
0.060	0.1556	0.7584	0.6706	0.9223

The two model representations remain strongly related across these durations, while their correspondence with the perceptual reference varies with scale. The initial half-trace convention belongs to the original orthonormal encoding; the revised calculation explicitly uses a fixed duration and reports its actual retention.

S6. Conditional stimulus-bootstrap intervals

Distances sharing a color are statistically dependent. We therefore resample color identities, following condition-resampling logic (Schütt et al., 2023). Each of 2,000 replicates draws 256 identities with replacement from the fresh ensemble, using PCG64 seed 20261010. The same draw is applied to every distance matrix. Upper-triangle comparisons between distinct original identities are retained, including multiplicities induced by repeated sampling; comparisons of an identity with itself are omitted. Spearman ranks are recomputed with average ranks for ties. Percentile endpoints are the 2.5th and 97.5th percentiles using linear interpolation.

The encoding, connectome, duration, neuronal parameters, and current normalization remain fixed. These intervals describe sensitivity to the composition of the new-color ensemble conditional on that model. They complement the held-out prediction analysis and time sensitivity. The paired difference is evaluated within each common resample.

S7. Relation to the initial encoding and its controls

The original orthonormal encoding gave CIEDE2000 correspondence 0.699493 at $t = 0.023$, compared with 0.860456 before propagation, and analytic–neuronal correspondence 0.944378. Those values describe the initial model on all 16 colors. They are distinct from the learned-model results and from averages over eight-color held-out subsets.

Table S4. New-color point estimates and conditional stimulus-bootstrap 95% intervals.

Comparison	Estimate	Interval
CIELAB–CIEDE2000	0.9229	$[0.9072, 0.9385]$
Learned propagated–CIEDE2000	0.9190	$[0.9014, 0.9361]$
Neuronal–CIEDE2000	0.8199	$[0.7945, 0.8465]$
Analytic–neuronal	0.9244	$[0.9098, 0.9382]$
Learned propagated minus CIELAB	-0.0039	$[-0.0154, 0.0075]$

The earlier 200 coefficient perturbations gave central 95% ranges $[0.657, 0.852]$ and $[0.920, 0.945]$ for perceptual-reference and analytic–neuronal correspondence. The bilaterally equal, centered support subspace remains fixed under these perturbations when the seeds are independent. Hence its orthonormal bases differ by an orthogonal transformation and the metric trace is invariant. This explains the identical selected times algebraically.

The original random-encoding ensembles contained 2,000 centered orthonormal bases on the same visual support and 2,000 on randomly selected eight-region supports. Median CIEDE2000 correlations were 0.841 and 0.846; 98.7% and 98.55%, respectively, exceeded the initial encoding's value. These comparisons describe that stipulated encoding relative to the sampled alternatives. Random support changes anatomical location, degree, and weighted strength together: the selected regions have mean degree 3.625, compared with 6.974 across the network. Thus the anatomical variables in that comparison are jointly changed.

An additional control preserves the individual degrees of the eight selected regions, with the other 68 degrees free to vary. Two thousand independently seeded edge-pair randomization runs retain 265 edges and the original weight multiset, reject loops and duplicate edges, and retain connected final graphs. Each run uses 50×265 proposals from the original graph. A proposal retains the occurrences of selected-region endpoints, resamples other endpoints from the remaining 68 regions, and randomly pairs the four endpoints. Vertex strengths can change. With the initial encoding held fixed, each graph's Laplacian is spectrally normalized and its duration selected on $0 : 0.001 : 0.150$ by the half-trace criterion. Median CIEDE2000 correspondence is 0.8084, with central range $[0.7494, 0.8592]$, and 1,999 of 2,000 values exceed 0.6995. Median analytic–neuronal correspondence is 0.9166, with range $[0.8919, 0.9364]$, and all sampled values lie below 0.9444. These results characterize two different properties of the initial encoding–network combination.

The revised model addresses the encoding choice through explicit estimation and new-color evaluation. Its metric factorization also clarifies the role of calibration: for any positive-definite G, the choice $F = G^{-1/2}H^{1/2}$ realizes the same fitted metric H. The learned-model results therefore concern the representation of color relations through the specified encoding and propagator. Independent anatomical constraints on encoding would

further determine the biological interpretation. The experimental comparisons proposed in Section 7 of the main manuscript concern subjective color judgements, measured neuronal responses, and estimated propagation geometry.

References

Heiko H. Schütt, Alexander D. Kipnis, Jörn Diedrichsen, and Nikolaus Kriegeskorte. Statistical inference on representational geometries. *eLife*, 12:e82566, 2023. doi: 10.7554/eLife.82566.

Gaurav Sharma, Wencheng Wu, and Edul N. Dalal. The CIEDE2000 color-difference formula: Implementation notes, supplementary test data, and mathematical observations. *Color Research & Application*, 30(1):21–30, 2005. doi: 10.1002/col.20070.

Institute for Cross-Disciplinary Physics and Complex Systems (IFISC), CSIC-UIB, Palma de Mallorca, Spain. E-mail: estrada@ifisc.uib-csic.es
